# Imine Reduction with Me_2_S-BH_3_

**DOI:** 10.3390/molecules26185443

**Published:** 2021-09-07

**Authors:** Mohammad M. Kamal, Zhizhou Liu, Siyuan Zhai, Dragoslav Vidović

**Affiliations:** 1 School of Chemistry, Faculty of Sciences, Monash University, Clayton 3800, Australia; mohammad.kamal@monash.edu (M.M.K.); liuzz@sibet.ac.cn (Z.L.); siyuan.zhai@monash.edu (S.Z.); 2 Suzhou Institute of Biomedical Engineering and Technology, Chinese Academy of Sciences, Suzhou 215163, China

**Keywords:** hydroboration, synthesis, aldimine, ketimine

## Abstract

Although there exists a variety of different catalysts for hydroboration of organic substrates such as aldehydes, ketones, imines, nitriles etc., recent evidence suggests that tetra-coordinate borohydride species, formed by activation, redistribution, or decomposition of boron reagents, are the true hydride donors. We then proposed that Me_2_S-BH_3_ could also act as a hydride donor for the reduction of various imines, as similar compounds have been observed to reduce carbonyl substrates. This boron reagent was shown to be an effective and chemoselective hydroboration reagent for a wide variety of imines.

## 1. Introduction

**Scheme 1:**
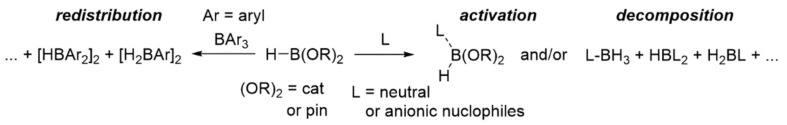
Observed activation, redistribution and/or decomposition of HBcat/HBpin in presence of nucleophiles (L) or other boranes (BAr_3_).

Hydroboration can be considered as one of the most powerful methods for reduction of various organic substrates such as aldehydes, ketones, imines, and nitriles under mild reaction conditions [1,2,3]. Pinacolborane (HBpin) or catecholborane (HBcat) has been predominantly used as the hydroborating agent in these particular transformations, but the boron fragment (i.e., Bcat/Bpin) was normally sacrificed to yield, for example, free alcohols (from carbonyls) or amines (from imines/nitriles). Furthermore, these reduction reactions were mainly performed in the presence of catalytic amounts of a diverse range of (in)organic/organometallic compounds [4,5,6,7,8,9]. Several of the described hydroboration reactions were efficient as catalyst loadings as low as 0.001 mol%, resulting in excellent substrate conversions [7]. Nevertheless, the exact role of these presumed (pre)catalytic species has been divisive, as several reports provided convincing evidence for the existence of hidden boron catalysis (HBC), i.e., the main role of the species that were introduced in “catalytic” amounts was the formation, via activation, redistribution, or decomposition (Scheme 1) of HBcat/HBipn, of boron-based compounds (e.g., hydroborates and boranes) that then acted as the true catalysts [10,11,12].

Four-coordinate borohydride compounds (e.g., HBR_3_^−^) were identified to serve as the (pre)catalysts for hydroboration of a hetero-atom containing unsaturated substrates such as aldehydes, ketones and imines [11,12]. This would then suggest that L-BH_3_ (L = THF, SMe_2_, NR_3_ (R = alkyl), *N*-heterocyclic carbene (NHC), etc.) could not only act as adequate reagents for reduction of these types of substrates but also deliver more cost-effective hydroboration protocol(s) as certain BH_3_-containing species act as synthetic precursors to HBpin/HBcat [13,14]. However, there appears to be a limited number of published works that use these particular reagents (i.e., L-BH_3_) for this specific purpose, with THF-BH_3_ being the preferred choice for hydroboration of mainly carbonyl substrates [15,16,17,18,19,20]. Furthermore, NHC-BH_3_ adducts were shown to be adequate reduction agents for C=X fragments (X=N, O, etc.), but an addition of an activator (e.g., silica gel, p-toluenesulfonic acid) was required [21,22]. The presence of protic activators (e.g., Al_2_O_3_) and/or protic solvent media (e.g., MeOH) were also required for efficient reduction of these substrates with, for example, NaBH_4_ and NaBH_3_CN [23,24,25,26,27]. Lastly, although ammonia borane (NH_3_-BH_3_) has been used for reduction of various aldehydes, ketones, imines, etc. [28,29,30,31], experimental and computational studies suggested that these particular reduction reactions underwent a concerted (double) hydrogen transfer mechanism [28,31], which is not typical for hydroboration reactions (see below). Therefore, herein we disclose chemoselective hydroboration of imines using solely Me_2_S-BH_3_ as the reducing agent in the absence of any activators and/or a protic solvent medium.

## 2. Results & Discussion

Instead of generating an “optimized” reaction condition with one of the examined imines and then implementing this procedure for the rest of the substrates, we decided to optimize each transformation in order to maximize the substrate conversions. Thus, the reactions were screened by varying the amount of Me_2_S-BH_3_ and the reaction temperature while the reactants were mixed in about 1 mL of CDCl_3_ in a sealed J. Young NMR tube. The most important outcomes and observations are summarized in Table 1. In a vast majority of examined transformations, heating to 60 °C was necessary to obtain quantitative substrates conversions with Me_2_S-BH_3_ loadings varying between 0.75 and 1.50 equiv. For example, most of the reaction mixtures showed negligible reactivity at room temperature, while imine substrates with enhanced steric properties (entries 5 and 6, Table 1) required, in general, 1.50 equiv loadings of Me_2_S-BH_3_ with respect to the imine. Reductions of imines that contain a 2,6-disopropylaniline fragment (e.g., entry 5) have been rarely examined, presumably due to low conversions of these particular substrates under the reported reaction conditions [32,33]. Furthermore, reduction of the imines that contained *N*-aryl substituents (entries 4 and 12) required not only a lower Me_2_S-BH_3_ loading (e.g., 0.75 vs. 1.10 mmol) but also a shorter reaction time (e.g., 6 vs. 12 h) in comparison to their *N*-alkyl containing analogues (e.g., entries 2 and 3 vs. entry 12). This can be potentially explained by the presence of the resonance structures involving the *N*-aryl fragment puling the electron density away from the N=C fragment and hence allowing hydride transfer to the carbon atom of this fragment (see below). It was then not surprising to observe that the presence of an electron withdrawing group (CF_3_) had a rate-enhancing effect (entry 7) while an electron donating group (OMe) had an opposite effect (entry 8). These observations suggested that the rate limiting step for the examined reactions was nucleophilic in nature (i.e., hydride transfer from a B-H fragment to the imine substrate; see below) and not electrophilic (i.e., formation of a imine-BH_3_ adduct) [20]. 

More importantly, according to ^1^H-NMR spectroscopy, all reactions resulted exclusively or solely in the anticipated reduction of the C=N double bond. This was particularly important for the reduction of the imine substrates that also contained an alkenyl group (i.e., α,β-unsaturated imines; entries 13 and 14, Table 1). Quantitative substrate conversions with excellent chemoselectivities (>98%) were achieved with these particular imines, while, at the same time, generating the fastest reaction rates among the examined substrates, despite the transformations performing at −78 °C (for the selectivity purposes). Lastly, according to the results summarized in Table 1, it appeared that this reduction protocol favored, in terms of reaction rates, ketimines over aldimines, which is not typically observed in the literature [10,32,33,34,35,36,37,38,39,40,41,42,43,44,45]. At the moment, the precise reason(s) for this observation is(are) not known but it may suggest that the electrophilic step i.e., coordination of imine to BH_3_ (see below) was rate determining, as one would expect that the hydride transfer (i.e., the nucleophilic step) would be less favored for ketimines over aldimines. 

**Scheme 2:**
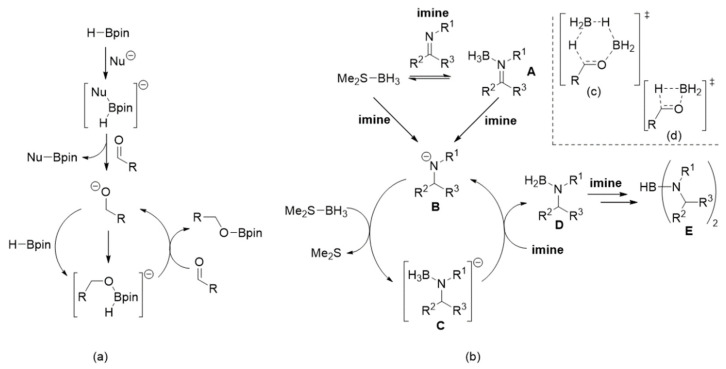
(**a**) The Clark mechanism; (**b**) the proposed mechanism for hydroboration of imines with MeS_2_-BH_3_; (**c**) and (**d**) proposed transition states for the hydride step in reduction of carbonyl compounds.

As mentioned in the introduction, catalytic hydroboration of unsaturated C=X fragments (X=O, N, etc., but X≠C) has been a controversial topic. However, there is a significant body of evidence suggesting that four-coordinate B-H containing compound(s) (usually anionic), generated by activation, decomposition, or redistribution of boron reagents, act as initial hydride donors and hence as initiators of catalytically active species [11,12]. Clark and co-workers suggested a mechanism (Scheme 2a) that involved “activation” of HBpin (or HBcat) by coordination of a nucleophile (the electrophilic step), followed by hydride transfer (the nucleophilic step) from boron to the substrate (e.g., aldehyde), to yield the corresponding anion (e.g., alkoxide) [46]. This anion would then bind to another molecule of H-Bpin to generate the catalytically active species (e.g., [HBpin(alkoxy)]^−^). Thus, we propose that for reduction of imines with Me_2_S-BH_3_, initial hydride transfer occurs from either Me_2_S-BH_3_ or imine-BH_3_ (**A**, Scheme 2b) to produce amide anion **B**. This anion then displaces Me_2_S from Me_2_S-BH_3_ to generate the catalytically active species **C**, which acts as a hydride donor to another imine completing the cycle while also yielding reduced species **D**.

**Figure 1:**
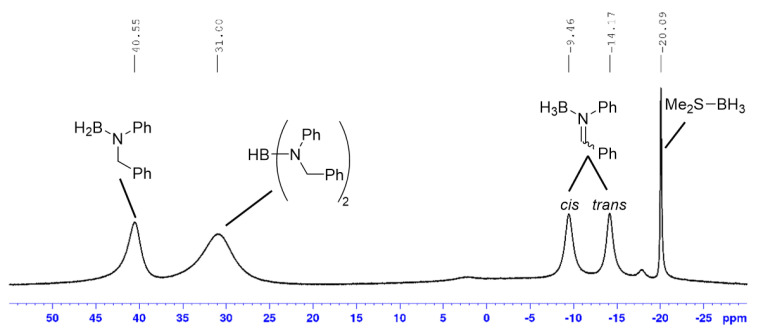
^11^B[^1^H]-NMR spectrum of a mixture (1:1) between Me_2_S-BH_3_ and *N*-benzylideneaniline after 6 h.

Recently, Abe and Yamataka proposed that reduction of carbonyl compounds using BH_3_ (in THF), the first step was H_3_B-carbonyl adduct formation (similar to **A**, Scheme 2b), followed by a hydride transfer step via a BH_3_-assisted transition state (Scheme 2c) [20]. However, although a majority of our examined hydroboration reactions require excess Me_2_S-BH_3_, it was still possible to achieve quantitative imine reduction with sub-stoichiometric amounts (0.75 mol%) of this boron reagent for several transformations (entries 1, 4, 13 and 14; Table 1). This suggested that, at least in certain instances, it was not only possible to reduce more than one imine substrate with one equivalent of Me_2_S-BH_3_ but also that the BH_3_-catalysed hydride transfer step (in our case going from **A** to **D**) step was less likely to occur. Furthermore, it was also suggested that the hydride transfer step (e.g., **A** → **D** in our case) occurred via a bimolecular transition state (Scheme 2d) [19]. This would help explain our observation that more than one equivalent of imine was reduced by MeS_2_-BH_3_ but a recent theoretical study indicated that a similar transition was high in energy [47]. Regardless of the nature of the hydride transfer step(s), it is still important to mention that we identified, via ^11^B[^1^H]-NMR spectroscopy, several proposed intermediates described in Scheme 2b. After mixing Me_2_S-BH_3_ and *N*-benzylideneaniline in a 1:1 mol ratio at room temperature for 6 h, it was possible to detect respective intermediates **A** (δ_B_ ~ −9 (*cis*) and −14 (*trans*) ppm, Figure 1; [48]), **D** (δ_B_ ~ 41 ppm; [49]) and **E** (δ_B_ ~ 31 ppm; [50,51]). The fact that unreacted Me_2_S-BH_3_ (δ_B_ ~ −20 ppm) was also present strongly suggested the existence of an equilibrium process between this reagent and intermediate **A** as indicated in Scheme 2b.

In conclusion, we have shown that Me_2_S-BH_3_ could also be used for reduction of a number of imines under mild reaction conditions and excellent chemoselectivity control. We have also managed to detect several key intermediates in the overall reaction pathway, which should aid in a better understanding of the overall hydroboration mechanism.

## 3. Materials and Methods

All imines were synthesized according to the literature reports (Table 2), while Me_2_S-BH_3_ was purchased from a commercial source and used as received. CDCl_3_ was dried by distilling it over CaSO_4_, while CH_2_Cl_2_ was dried by distilling over CaH_2_. Reduction of imines was performed using standard Schlenk techniques, while subsequent work-up steps (with MeOH) were performed in on a benchtop.

General procedure for reduction of imines: After 1.0 mmol of an imine (entries 1–12) and Me_2_S-BH_3_ (amounts according to Table 1) were mixed in a sealed J. Young NMR tube using about 1 mL of CDCl_3_, the reaction mixture was left at 60 °C for the time duration indicated in Table 1. For α,β-unsaturated imines (entries 13 and 14), the reactants were mixed in CH_2_Cl_2_ at −78 °C. After the reaction was completed (via ^1^H-NMR spectroscopy), it was quenched with 5 mL of MeOH, followed by removal of all volatiles under reduced pressure. The crude product mixture was then dissolved in 10 mL ethyl acetate, washed three times with 10 mL of water/brine, and dried with MgSO_4_. All amine samples were collected as oils after removal of solvent apart from benzylmethylamine (entry 1) and N-benzylaniline (entry 4), which were obtained as solids. The spectroscopic data for all amines matched those reported (Table 2). 

Purity was assessed by ^1^H and ^13^C[^1^H]-NMR spectroscopy and all samples were >95% pure. ^1^H (400.2 MHz), ^13^C[^1^H] (100.6 MHz), and/or ^11^B[^1^H] (128.6 MHz) NMR spectra of reactions and/or isolated amines in CDCl_3_ were recorded on a Bruker Avance III 400. 

^1^H and ^13^C[^1^H]-NMR spectroscopic data for isolated amines (Supplementary Materials):

*N*-methyl-1-phenylmethanamine (Entry 1): ^1^H-NMR (400.2 MHz, CDCl_3_): *δ* 7.31 (m, 2H), 7.25 (m, 3H), 3.86 (s, 2H), 3.05 (s, br, 1H), 2.32 (s, 1H), 2.31 (s, 2H). ^13^C-NMR (CDCl_3_, 100.6 MHz): *δ* 136.5, 136.3, 129.8, 129.5, 128.3, 128.2, 127.7, 127.7, 66.8, 66.2, 48.4, 47.7.

*N*-benzylpropan-2-amine (Entry 2): ^1^H-NMR (400.2 MHz, CDCl_3_): *δ* 7.25 (m, 4H), 7.17 (m, 1H), 3.71 (s, 2H), 2.79 (hept, ^3^*J* = 6.2 Hz, 1H), 1.57 (s, br, 1H), 1.03 (d, ^3^*J* = 6.2 Hz, 6H). ^13^C-NMR (CDCl_3_, 100.6 MHz): *δ* 140.6, 128.4, 128.2, 126.9, 51.6, 48.1, 22.9.

*N*-benzyl-2-methylpropan-2-amine (Entry 3): ^1^H-NMR (400.2 MHz, CDCl_3_): *δ* 7.25 (m, 4H), 7.15 (m, 1H), 3.66 (s, 2H), 1.11 (s, 9H). ^13^C-NMR (CDCl_3_, 100.6 MHz): *δ* 140.1, 127.4, 127.3, 125.8, 49.9, 46.2, 28.0, 27.3.

*N*-benzylaniline (Entry 4): ^1^H-NMR (400.2 MHz, CDCl_3_): *δ* 7.26 (m, 5H), 7.10 (m, 2H), 6.67 (m, 1H), 6.59 (m, 2H), 4.64 (s, br, 1H), 4.25 (s, 2H). ^13^C NMR (CDCl_3_, 100.6 MHz): *δ* 146.4, 137.9, 128.3, 127.6, 126.7, 126.3, 117.1, 112.4, 47.7.

*N*-benzyl-2,6-diisopropylaniline (Entry 5): ^1^H-NMR (400.2 MHz, CDCl_3_): *δ* 7.29 (m, 5H), 7.04 (m, 3H), 4.00 (s, 2H), 3.24 (hept, ^3^*J* = 6.8 Hz, 2H), 1.16 (d, ^3^*J* = 6.8Hz, 12H). ^13^C NMR (CDCl_3_, 100.6 MHz): *δ* 142.9, 128.6, 128.1, 127.5, 123.7, 56.0, 27.8, 24.3.

*N*-(2,6-dimethylbenzyl)propan-2-amine (Entry 6): ^1^H-NMR (400.2 MHz, CDCl_3_): *δ* 6.93 (m, 3H), 3.66 (s, 2H), 2.84 (hept, ^3^*J* = 6.2 Hz, 1H), 2.32 (s, 6H), 1.05 (d, ^3^*J* = 6.2 Hz, 6H). ^13^C-NMR (CDCl_3_, 100.6 MHz): *δ* 135.9, 127.2, 125.9, 48.6, 44.6, 21.9, 18.5.

*N*-(4-(trifluoromethyl)benzyl)propan-2-amine (Entry 7): ^1^H-NMR (400.2 MHz, CDCl_3_): *δ* 7.59 (d, ^3^*J* = 8.0 Hz, 2H), 7.47 (d, ^3^*J* = 8.0 Hz, 2H), 3.86 (s, 2H), 2.87 (hept, ^3^*J* = 6.2 Hz, 1H), 1.78 (s, br. 1H), 1.13 (d, ^3^*J* = 6.2Hz, 6H). ^13^C-NMR (CDCl_3_, 100.6 MHz): *δ* 144.7, 129.3, 129.0, 128.3, 125.6, 125.3, 122.9, 50.9, 48.2, 22.8.

*N*-(4-methoxybenzyl)propan-2-amine (Entry 8): ^1^H-NMR (400.2 MHz, CDCl_3_): *δ* 7.18 (d, ^3^*J* = 8.4 Hz, 2H), 6.78 (d, ^3^*J* = 8.4 Hz, 2H), 3.71 (s, 3H), 3.64 (s, 2H), 2.78 (hept, ^3^*J* = 6.2 Hz, 1H), 1.66 (s, broad, 1H), 1.02 (d, ^3^*J* = 6.2 Hz, 6H). ^13^C-NMR (CDCl_3_, 100.6 MHz): *δ* 158.6, 132.6, 129.4, 113.8, 55.3, 50.9, 48.0, 22.8.

*N*-benzhydrylpropan-2-amine (Entry 9): ^1^H-NMR (400.2 MHz, CDCl_3_): *δ* 7.21 (m, 10H), 4.89 (s, 1H), 2.67 (hept, ^3^*J* = 6.2 Hz, 1H), 1.40 (s, br, 1H), 1.00 (d, ^3^*J* = 6.2 Hz, 6H). ^13^C-NMR (CDCl_3_, 100.6 MHz): *δ* 144.5, 128.4, 127.4, 126.9, 64.3, 46.2, 23.1. 

*N*-(1-phenylethyl)propan-2-amine (Entry 10): ^1^H-NMR (400.2 MHz, CDCl_3_): *δ* 7.20 (m, 4H), 7.13 (m, 1H), 3.80 (q, ^3^*J* = 6.4 Hz, 1H), 2.54 (hept, ^3^*J* = 6.2 Hz, 1H), 1.25 (d, ^3^*J* = 6.4 Hz, 3H), 0.94 (d, ^3^*J* = 6.2 Hz, 3H), 0.91 (d, ^3^*J* = 6.2 Hz, 3H). ^13^C-NMR (CDCl_3_, 100.6 MHz): *δ* 145.1, 127.4, 125.7, 125.4, 54.0, 44.5, 23.8, 23.0, 21.1.

*N*-isopropylcyclohexanamine (Entry 11): ^1^H-NMR (400.2 MHz, CDCl_3_): *δ* 2.89 (hept, ^3^*J* = 6.2 Hz, 1H), 2.43 (m, 1H), 1.81 (m, 2H), 1.65 (m, 2H), 1.54 (m, 2H), 1.10 (m, 4H), 0.97 (d, ^3^*J* = 6.2 Hz, 6H), 0.95 (m, 1H). ^13^C-NMR (CDCl_3_, 100.6 MHz): *δ* 53.5, 44.8, 34.0, 26.2, 25.3, 23.4.

*N*-cyclohexylaniline (Entry 12): ^1^H-NMR (400.2 MHz, CDCl_3_): *δ* 7.08 (t, ^3^*J* = 7.8 Hz, 2H), 6.55 (m, 3H), 3.75 (br s, 1H), 3.17 (m, 1H), 1.98 (m, 2H), 1.67 (m, 2H), 1.57 (m, 1H), 1.27 (m, 2H), 1.10 (m, 3H). ^13^C-NMR (CDCl_3_, 100.6 MHz): *δ* 147.1, 129.3, 117.1, 113, 4, 52.0, 33.4, 25.9, 25.0.

(*E*)-*N*-isopropyl-3-phenylprop-2-en-1-amine (Entry 13): ^1^H-NMR (400.2 MHz, CDCl_3_): *δ* 7.24 (m, 5H, Phenyl), 6.50 (d, *^3^J* = 15.8 Hz, 1H), 6.29 (dt, *^3^J* = 15.8 Hz, *^3^J* = 6.4 Hz, 1H), 3.39 (d, *^3^J* = 6.4 Hz, 2H), 2.87 (hept, *^3^J* = 6.2 Hz, 1H), 1.39 (s, broad, 1H), 1.08 (d, *^3^J* = 6.2Hz, 6H). ^13^C-NMR (CDCl_3_, 100.6 MHz): *δ* 137.1, 131.1, 128.6, 128.5, 127.3, 126.2, 48.4, 48.1, 22.9. 

(*E*)-*N*-isopropyl-1,3-diphenylprop-2-en-1-amine (Entry 14): ^1^H-NMR (400.2 MHz, CDCl_3_): *δ* 7.26 (m, 10H, phenyl), 6.53 (d, *^3^J* = 15.8 Hz, 1H), 6.31 (dd, *^3^J* = 15.8 Hz, *^3^J* = 7.3 Hz, 1H), 4.51 (d, *^3^J* = 7.3 Hz, 1H), 2.83 (hept, *^3^J* = 6.2 Hz, 1H), 1.11 (d, *^3^J* = 6.2 Hz, 3H), 1.08 (d, *^3^J* = 6.2 Hz, 3H). ^13^C-NMR (CDCl_3_, 100.6 MHz): *δ* 143.1, 136.98, 132.9, 130.0, 128.5, 128.4, 127.4, 127.3, 127.1, 126.4, 62.4, 45.6, 23.2, 22.8.

## Figures and Tables

**Table 1:** Summary of reaction conditions to achieve quantitative conversion of imines ^1^.

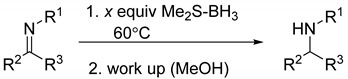						
**Entry**	**R^1^**	**R^2^**	**R^3^**	**Me_2_S-BH_3_ (Equiv)**	**Time (h)**	**Conversion (Isolated Yields)**
1 ^2^	Me	Ph	H	0.75	30	>99% (65%)
2	*^i^*Pr	Ph	H	1.10	72	>99% (81%)
3 ^3^	*^t^*Bu	Ph	H	1.10	24	>99% (71%)
4	Ph	Ph	H	0.75	2	>99% (87%)
5 ^3^	2,6-*^i^*Pr_2_-C_6_H_3_	Ph	H	1.50	6	>99% (88%)
6	*^i^*Pr	2,6-Me_2_-C_6_H_3_	H	1.50	12	>99% (84%)
7	*^i^*Pr	4-CF_3_-C_6_H_4_	H	1.10	30	>99% (76%)
8	*^i^*Pr	4-OMe-C_6_H_4_	H	1.10	96	>99% (80%)
9 ^3^	*^i^*Pr	Ph	Ph	1.10	18	>99% (44%)
10	*^i^*Pr	Ph	Me	1.10	6	>99% (61%)
11	*^i^*Pr	(CH_2_)_5_		1.10	12	>99% (52%)
12	Ph	(CH_2_)_5_		0.75	6	>99% (81%)
13 ^3,4^	*^i^*Pr	CH=CHPh (trans)	H	0.75	1	>99% (78%)
14 ^4^	*^i^*Pr	CH=CHPh (trans)	Me	0.75	1	>99% (65%)

^1^ Reactions were performed using 1.0 mmol of imines. ^2^ This reaction was also preformed using 1.0 g (8.4 mmol), resulting in 66% product yield. ^3^ These reactions were performed at least three times. ^4^ Reaction performed at −78 °C in DCM.

**Table 2:** Literature references for the synthesis of the examined imines and spectroscopic data for the corresponding amines.

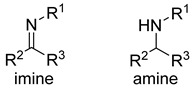					
**Entry**	**R^1^**	**R^2^**	**R^3^**	**References**	
				**Synthesis of Imines**	**Spectroscopic Data for Amines**
1	Me	Ph	H	[52]	[53]
2	*^i^*Pr	Ph	H	[52]	[37]
3	*^t^*Bu	Ph	H	[33,52]	[32]
4	Ph	Ph	H	[33,52]	[37,53]
5	2,6-*^i^*Pr_2_-C_6_H_3_	Ph	H	[33]	[54]
6	*^i^*Pr	2,6-Me_2_-C_6_H_3_	H	[52]	[54]
7	*^i^*Pr	4-CF_3_-C_6_H_4_	H	[55]	[37]
8	*^i^*Pr	4-OMe-C_6_H_4_	H	[52]	[37]
9	*^i^*Pr	Ph	Ph	[33,55]	[56]
10	*^i^*Pr	Ph	Me	[33]	[57]
11	*^i^*Pr	(CH_2_)_5_		[58]	[59]
12	Ph	(CH_2_)_5_		[58]	[59]
13	*^i^*Pr	CH=CHPh (trans)	H	[34,52,55]	[34,60]
14	*^i^*Pr	CH=CHPh (trans)	Me	[55]	[60]

## Data Availability

Raw data for this study are not required to be submitted to any archived datasets.

## Supplementary Materials

This information is available online. Copies of ^1^H and ^13^C-NMR spectra of isolated amines (Figures S1–S28).
