# Supplementary material for: Imine Reduction with Me2S-BH3
Source: Molecules. 2021 Sep 7;26(18):5443. doi: 10.3390/molecules26185443 (PMC8467297; doi:10.3390/molecules26185443)
Supplement: Supplementary file 1 [file molecules-26-05443-s001.zip › molecules-1300043-supplementary.pdf]

Supporting Information

Imine reduction with Me_2S-BH_3

Mohammad M. Kamal [1], Zhizhou Liu [1,2], Siyuan Zhai [1], Dragoslav Vidović [1,*]

1 School of Chemistry, Faculty of Sciences, Monash University, 3800 Clayton, Australia.; mohammad.kamal@monash.edu (M.K.); siyuan.zhai@monash.edu (S.Z)
2 Suzhou Institute of Biomedical Engineering and Technology, Chinese Academy of Sciences, Suzhou 215163, People's Republic of China; liuzz@sibet.ac.cn
* Correspondence: drasko.vidovic@monash.edu

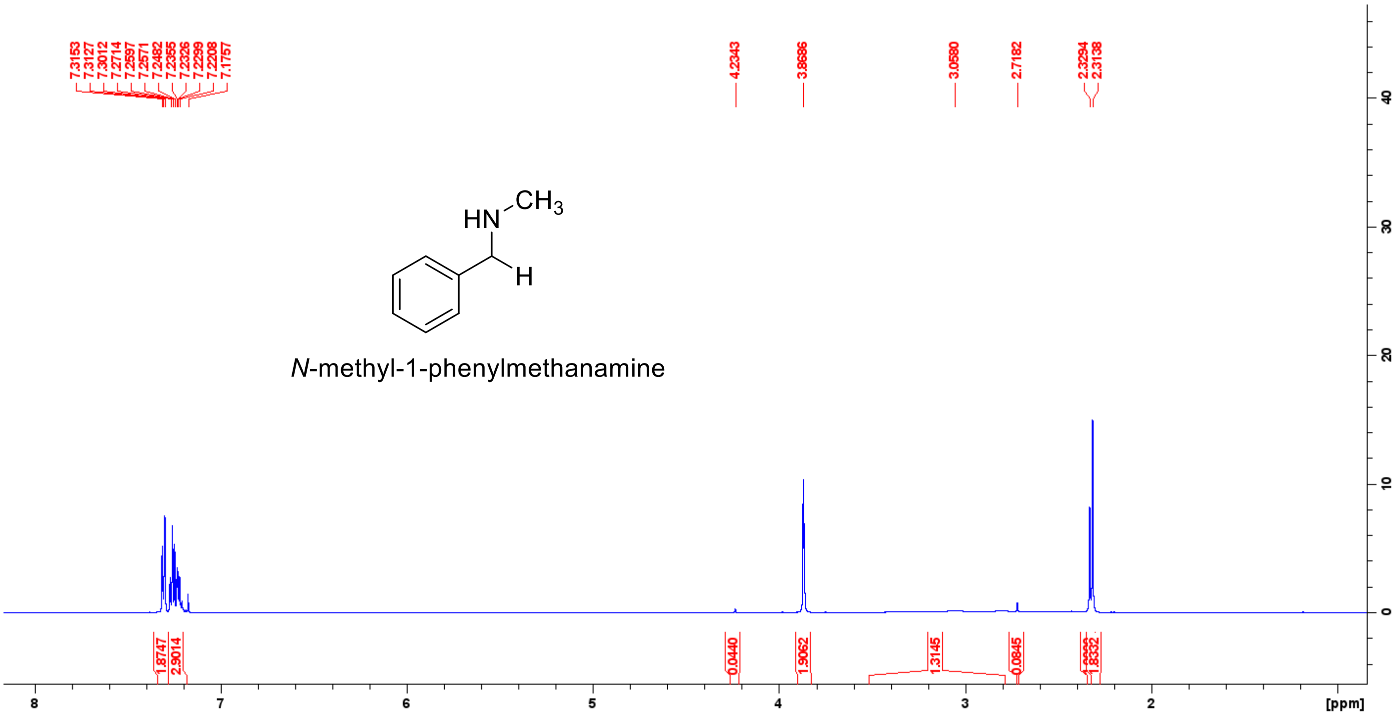

Figure S1. ^{1}H NMR spectrum for *N*-methyl-1-phenylmethanamine.

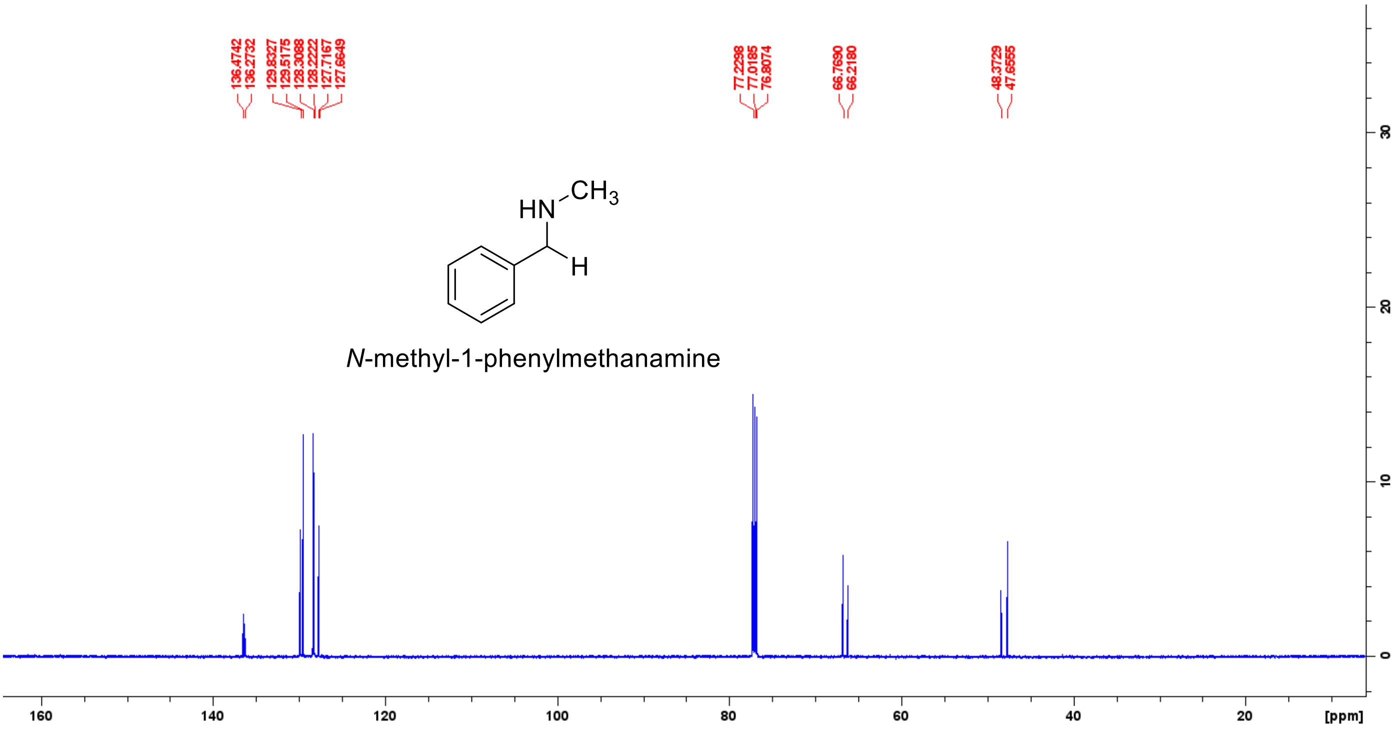

Figure S2. ^{13}C NMR spectrum for *N*-methyl-1-phenylmethanamine.

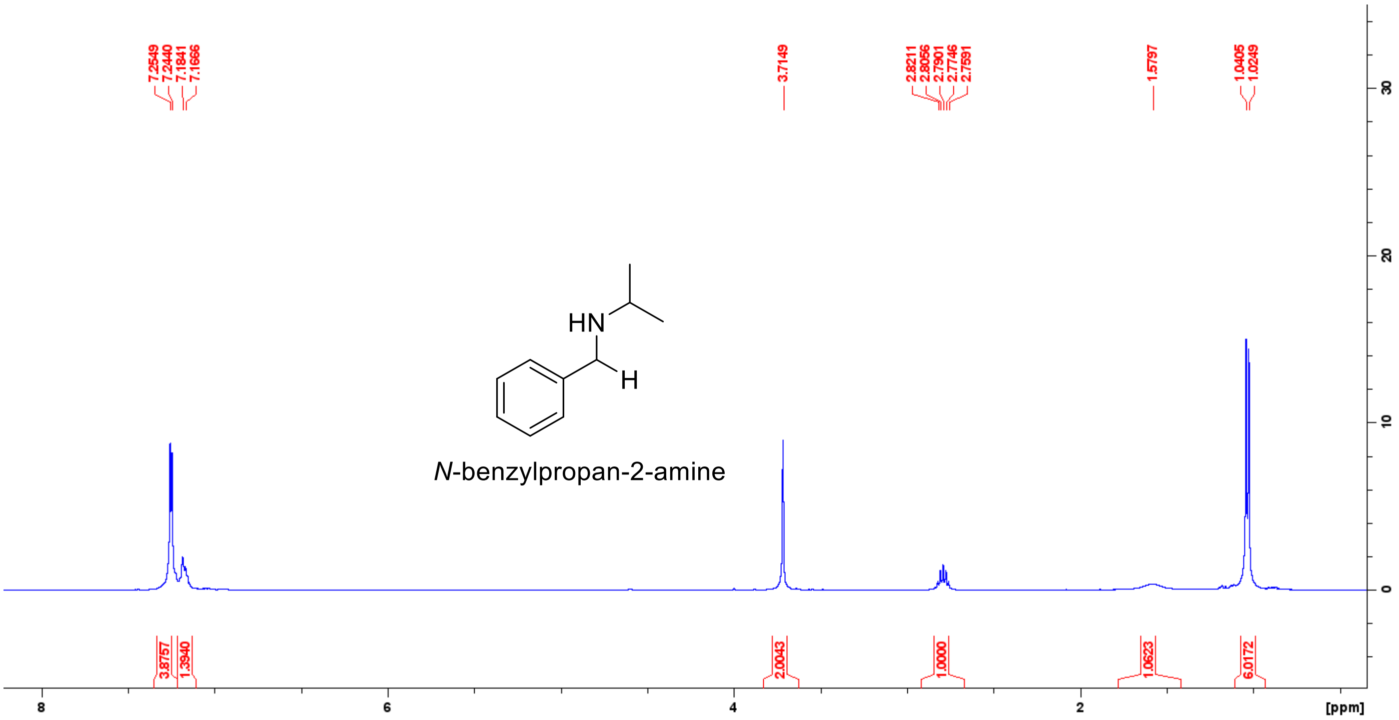

Figure S3. ^{1}H NMR spectrum for *N*-benzylpropan-2-amine.

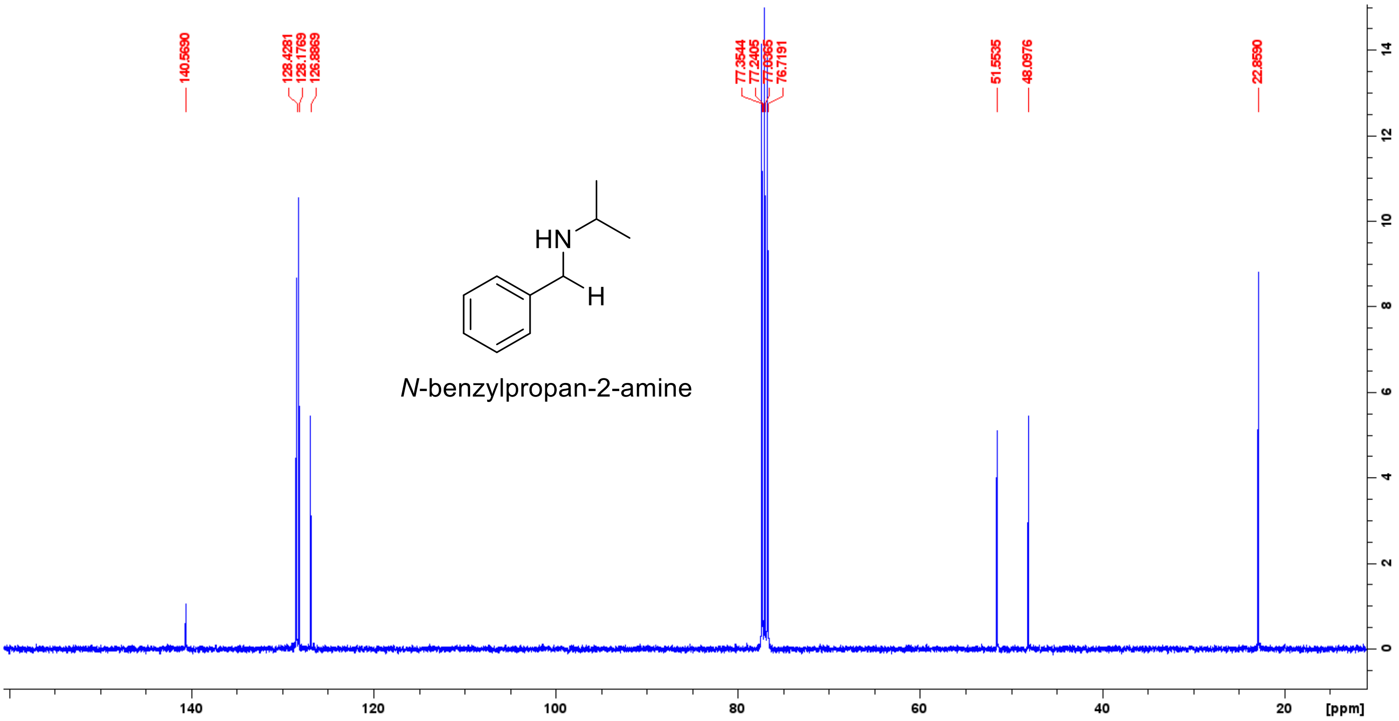

Figure S4. ^{13}C NMR spectrum for *N*-benzylpropan-2-amine.

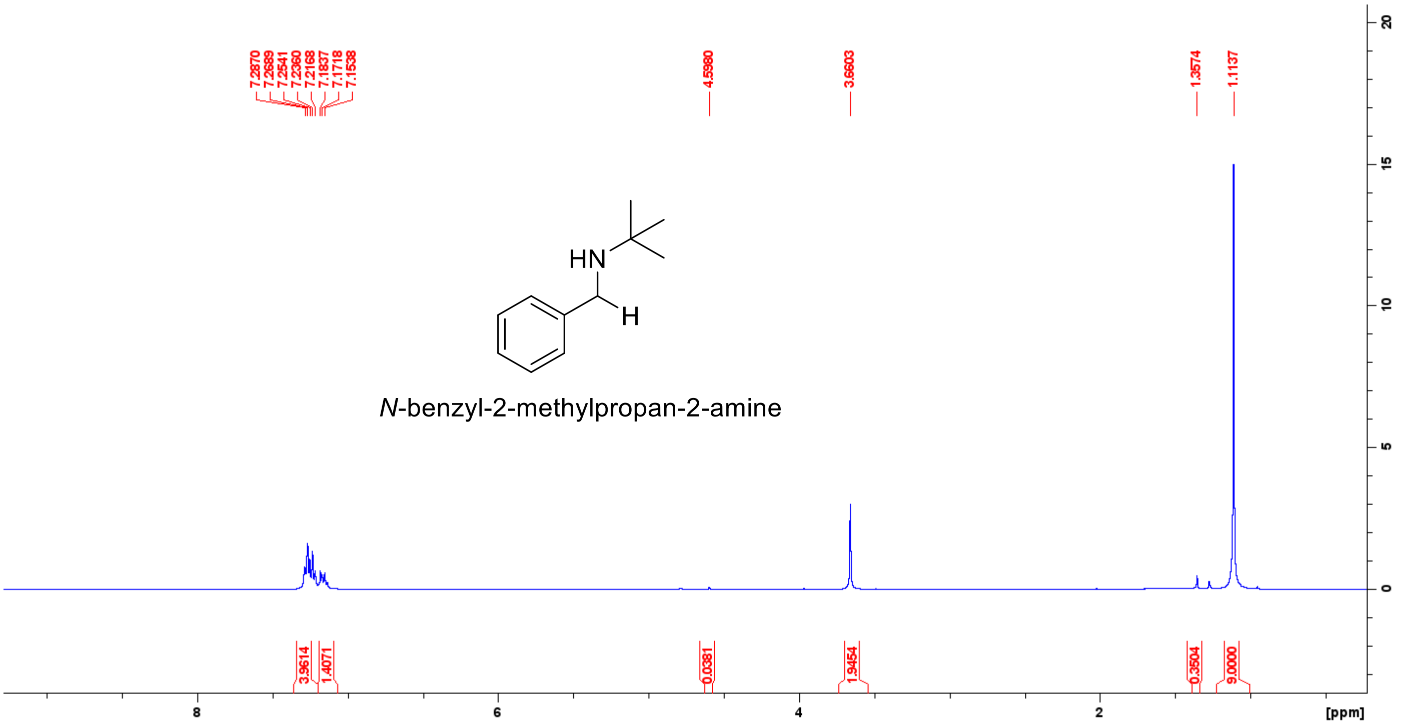

Figure S5. ^{1}H NMR spectrum for *N*-benzyl-2-methylpropan-2-amine.

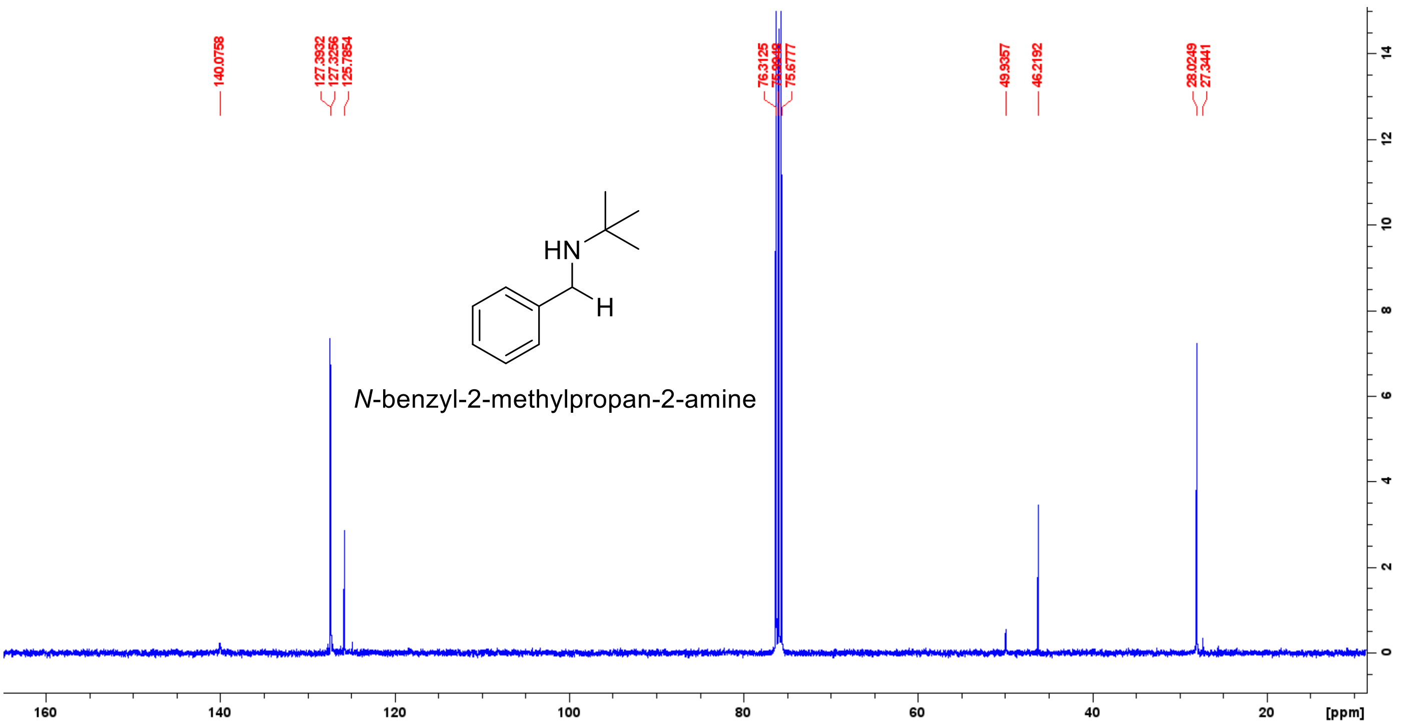

Figure S6. ^{13}C NMR spectrum for *N*-benzyl-2-methylpropan-2-amine.

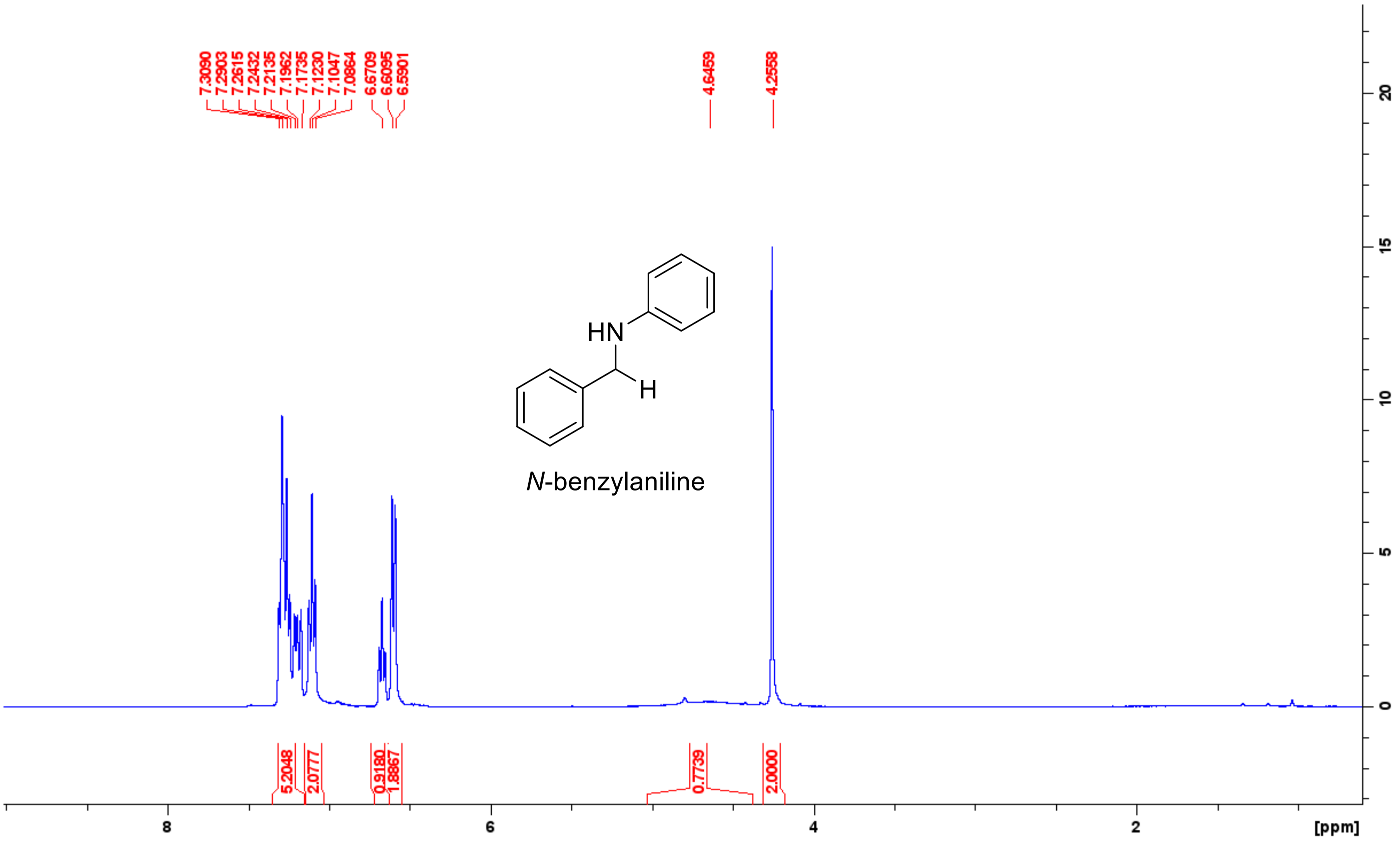

Figure S7. ^{1}H NMR spectrum for *N*-benzylaniline.

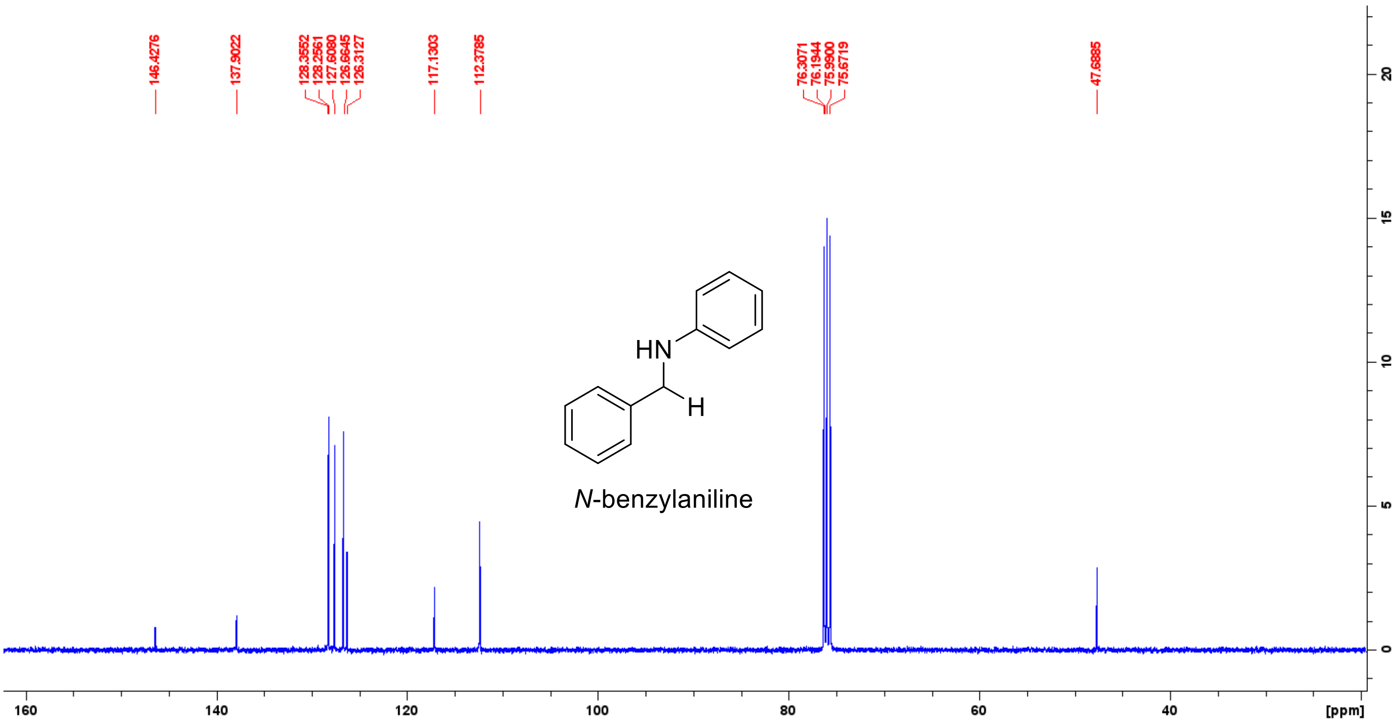

Figure S8. ^{13}C NMR spectrum for *N*-benzylaniline.

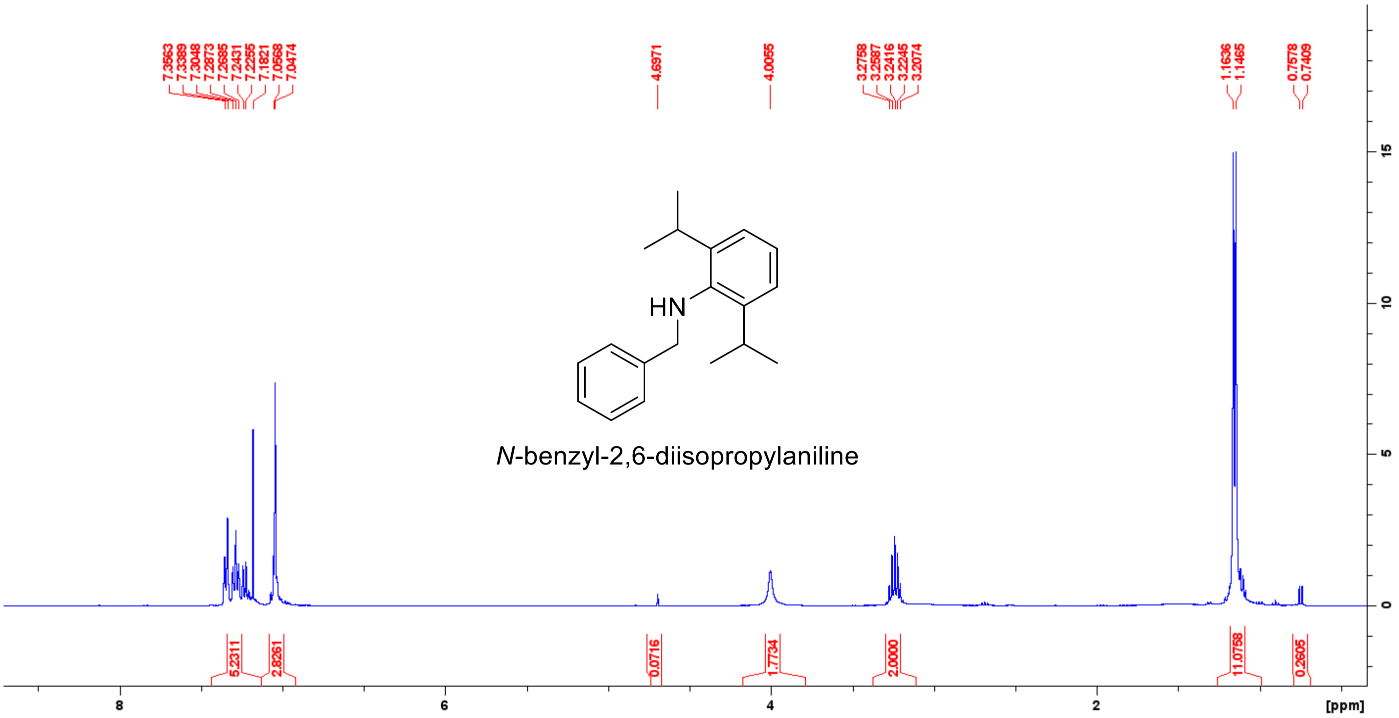

Figure S9. ^{1}H NMR spectrum for *N*-benzyl-2,6-diisopropylaniline.

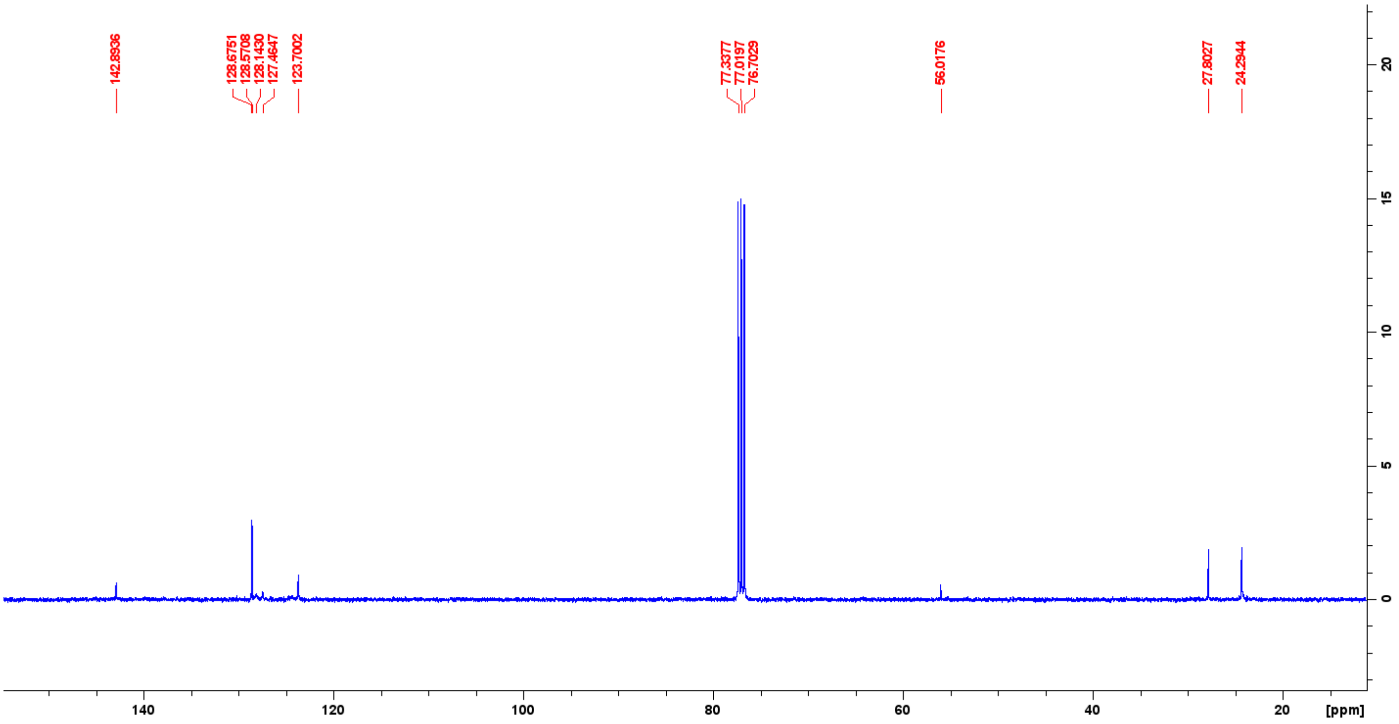

Figure S10. ^{13}C NMR spectrum for *N*-benzyl-2,6-diisopropylaniline.

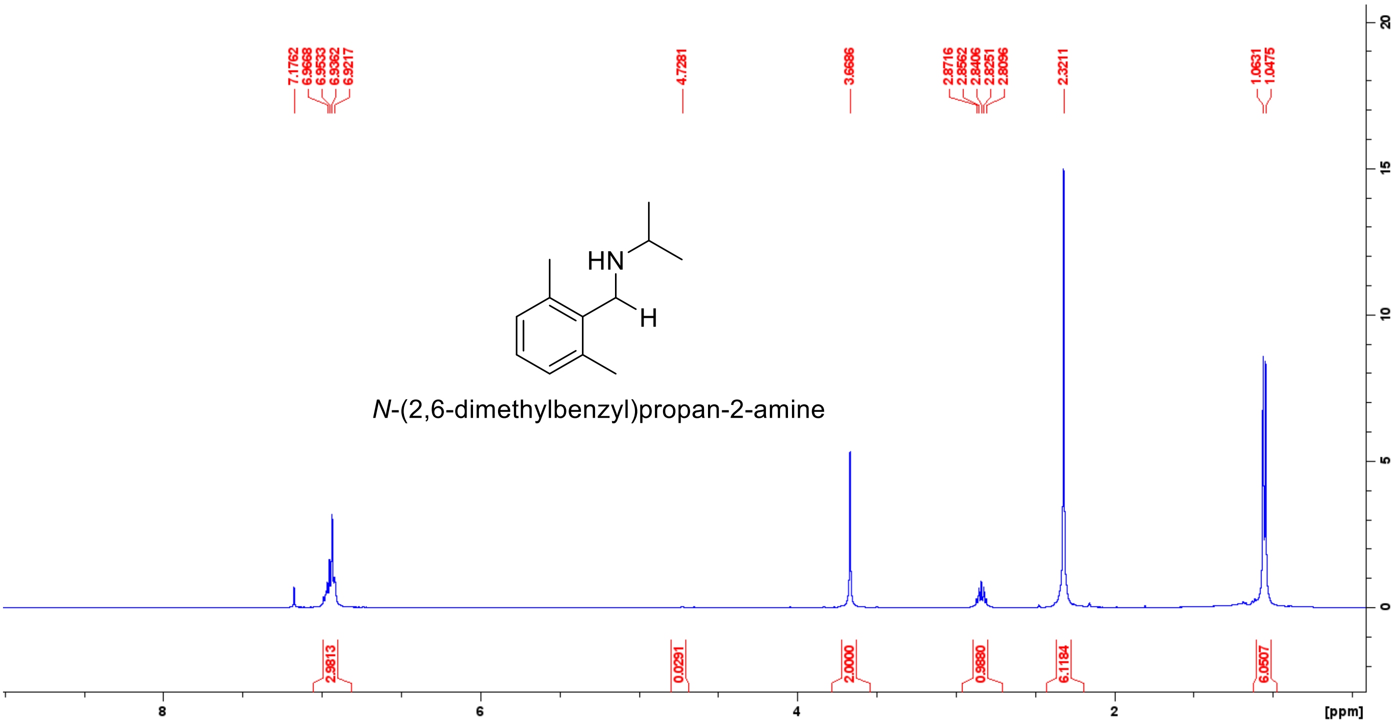

Figure S11. ^{1}H NMR spectrum for *N*-(2,6-dimethylbenzyl)propan-2-amine.

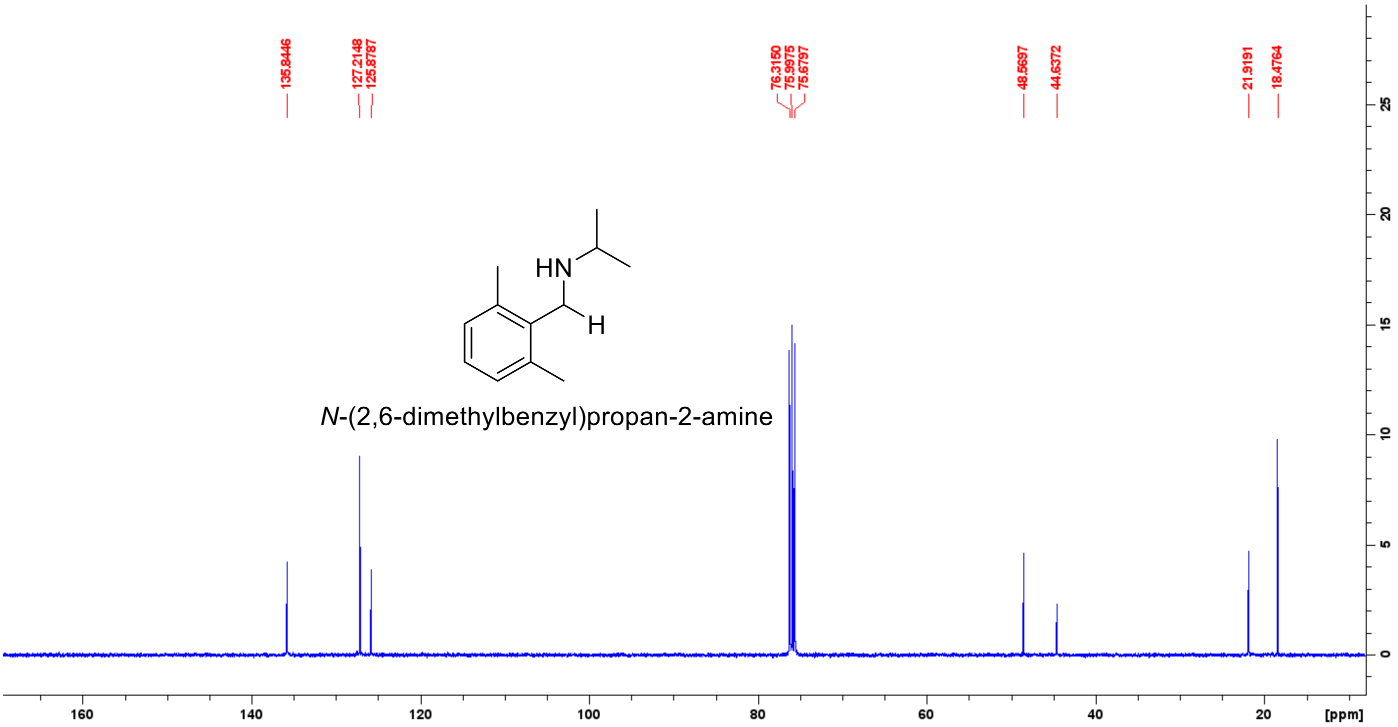

Figure S12. ^{13}C NMR spectrum for *N*-(2,6-dimethylbenzyl)propan-2-amine.

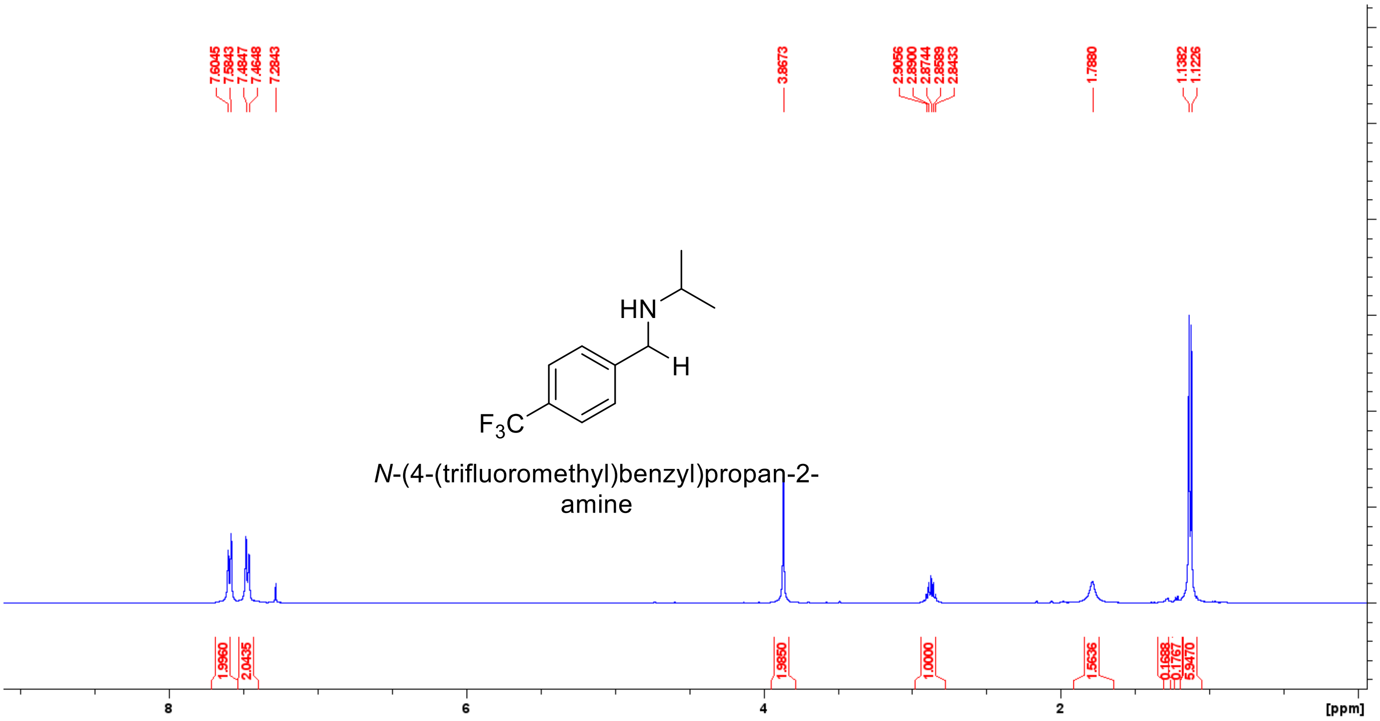

Figure S13. ^{1}H NMR spectrum for *N*-(4-(trifluoromethyl)benzyl)propan-2-amine.

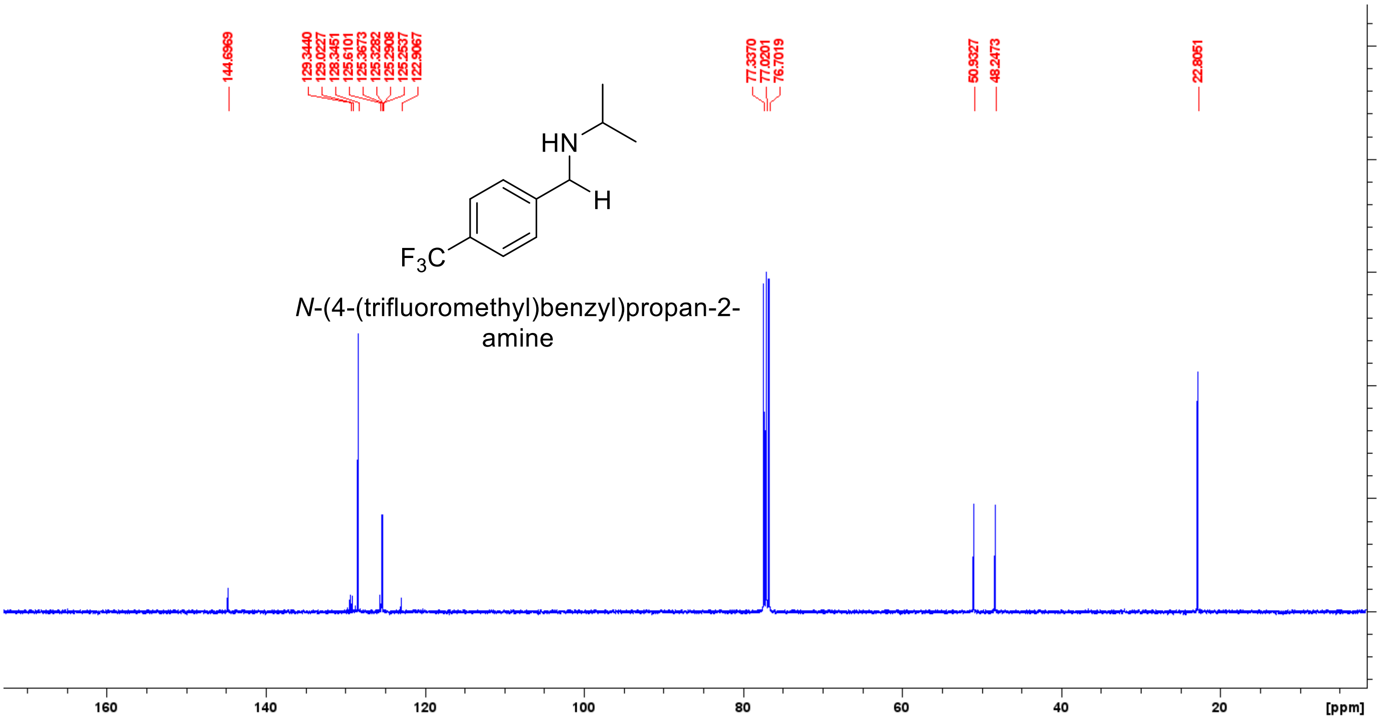

Figure S14. ^{13}C NMR spectrum for *N*-(4-(trifluoromethyl)benzyl)propan-2-amine.

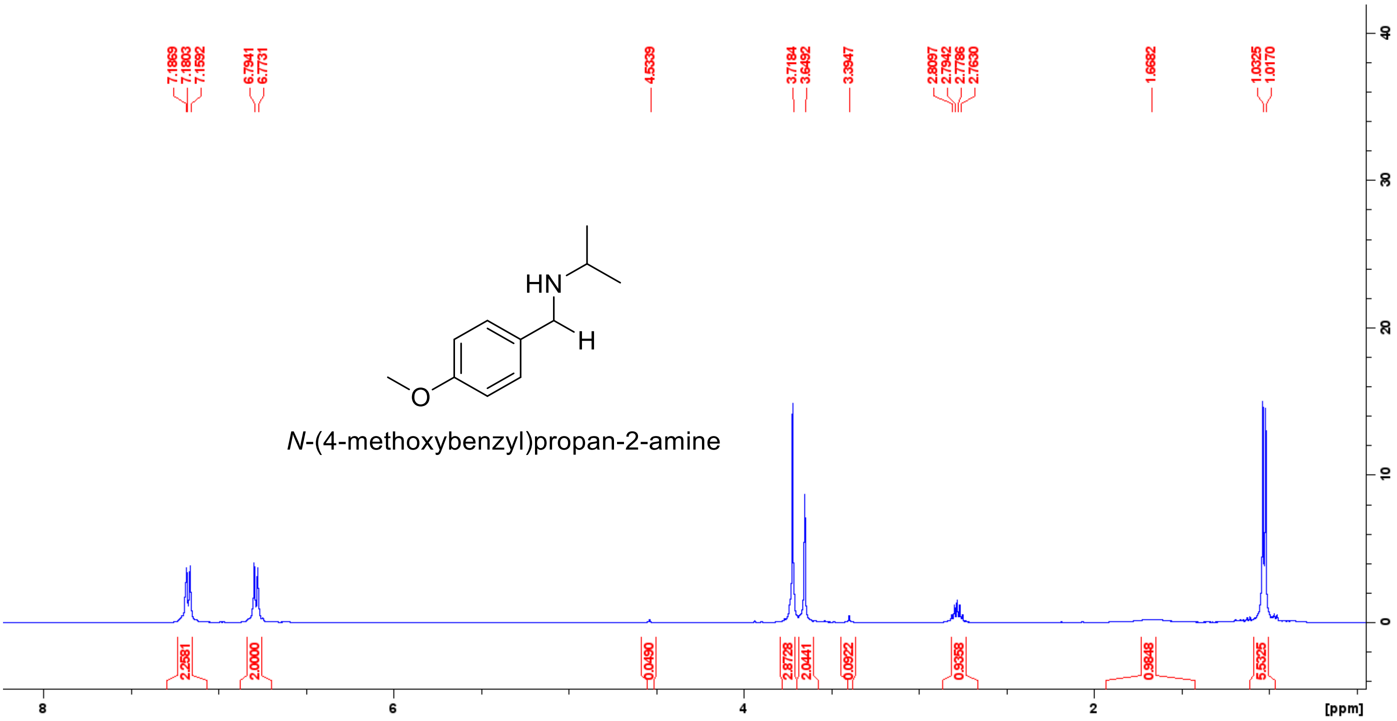

Figure S15. ^{1}H NMR spectrum for *N*-(4-methoxybenzyl)propan-2-amine.

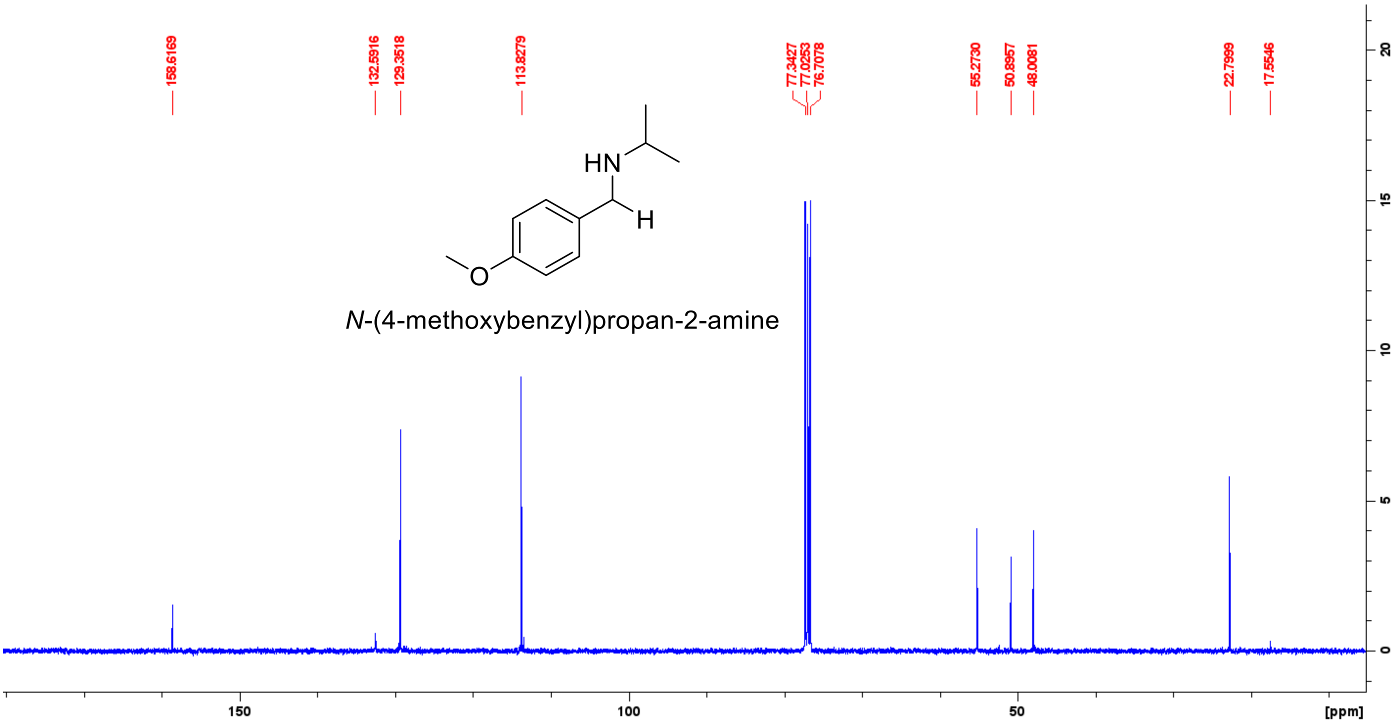

Figure S16. ^{13}C NMR spectrum for *N*-(4-methoxybenzyl)propan-2-amine.

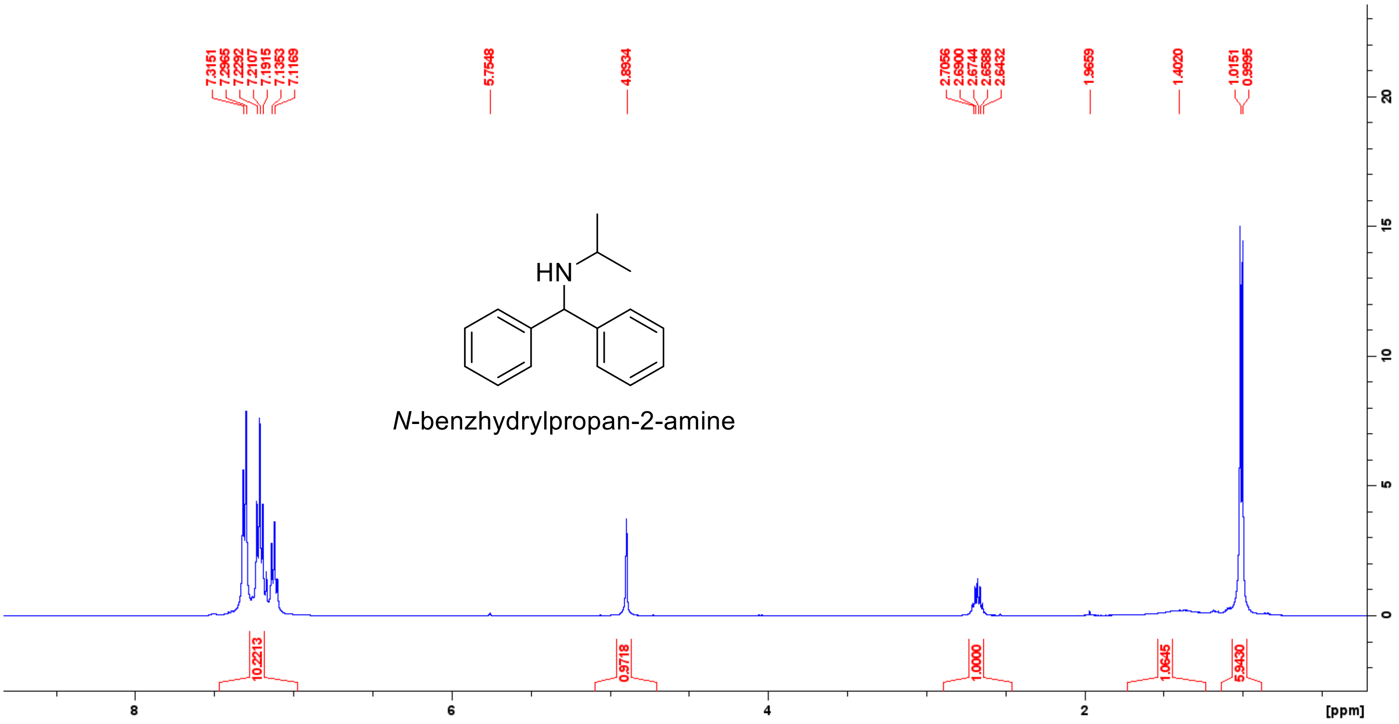

Figure S17. ^{1}H NMR spectrum for *N*-benzhydrylpropan-2-amine.

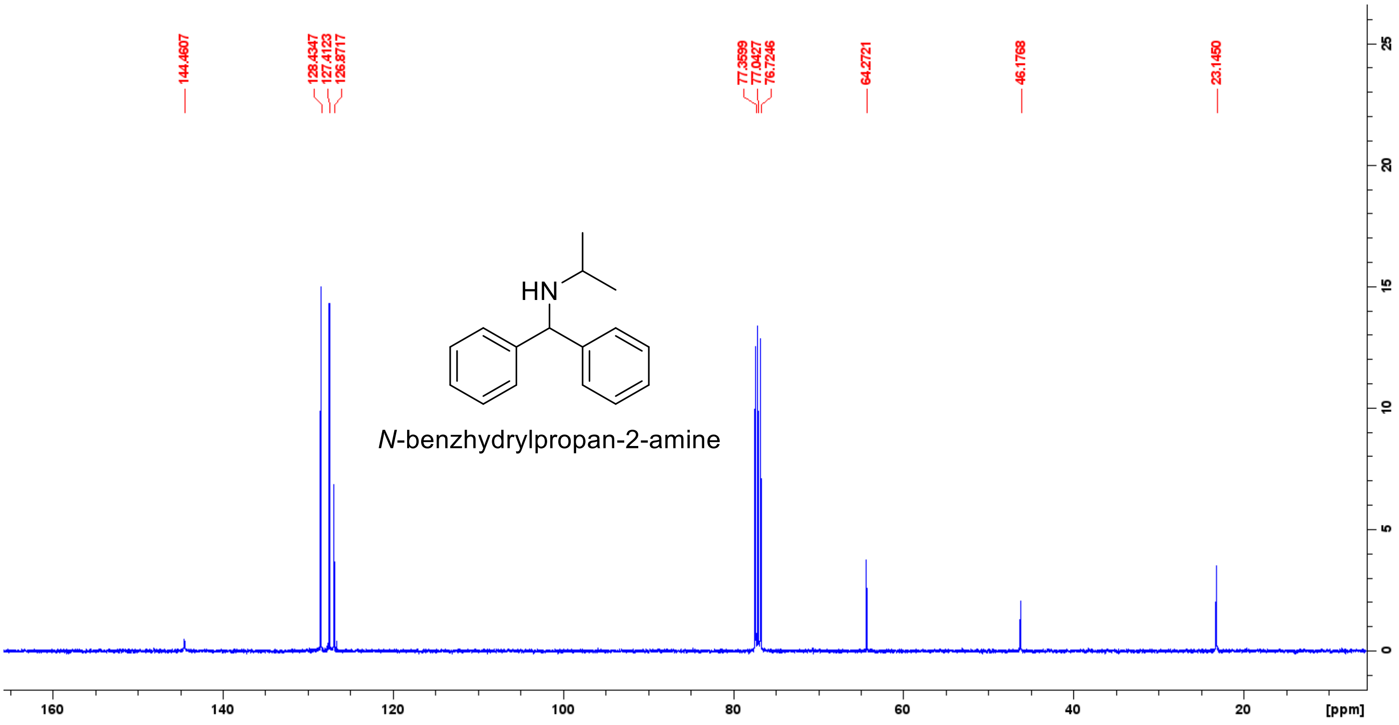

Figure S18. ^{13}C NMR spectrum for *N*-benzhydrylpropan-2-amine.

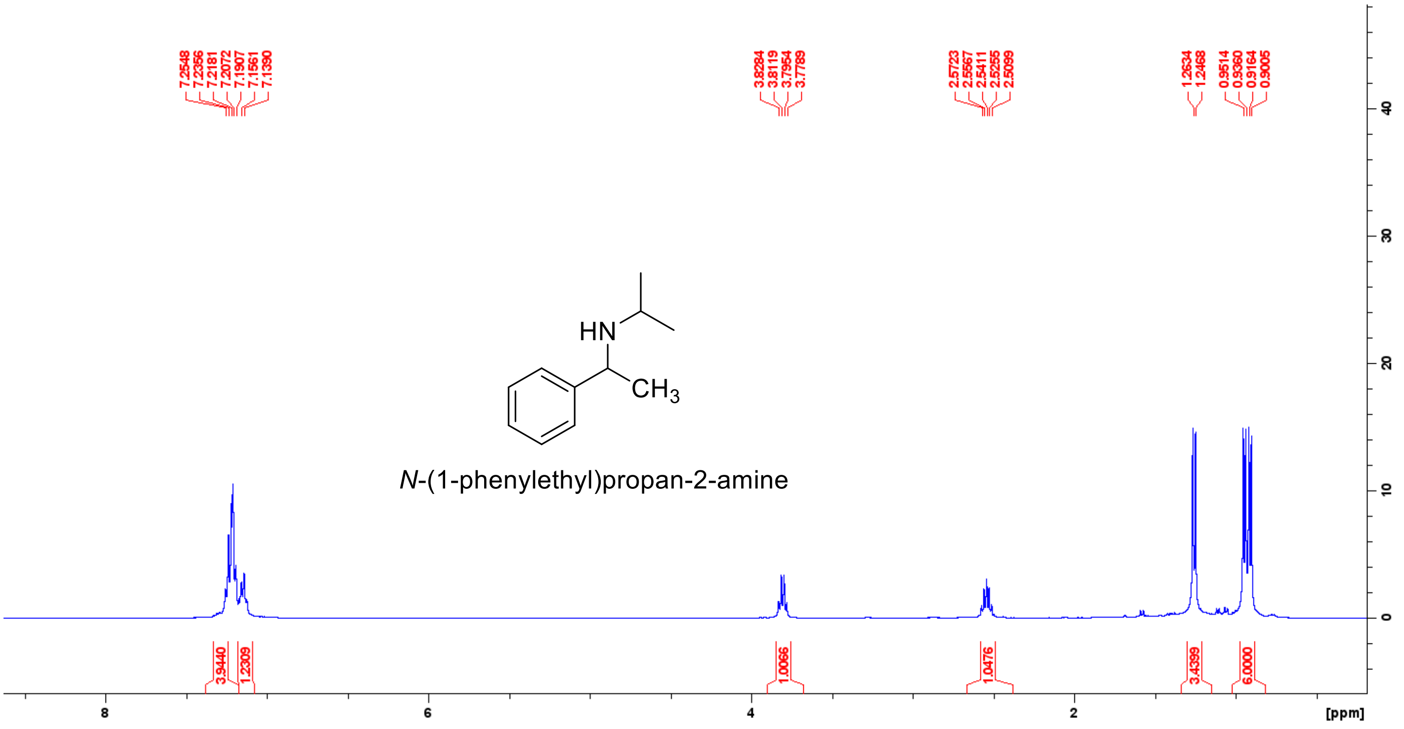

Figure S19. ^{1}H NMR spectrum for *N*-(1-phenylethyl)propan-2-amine.

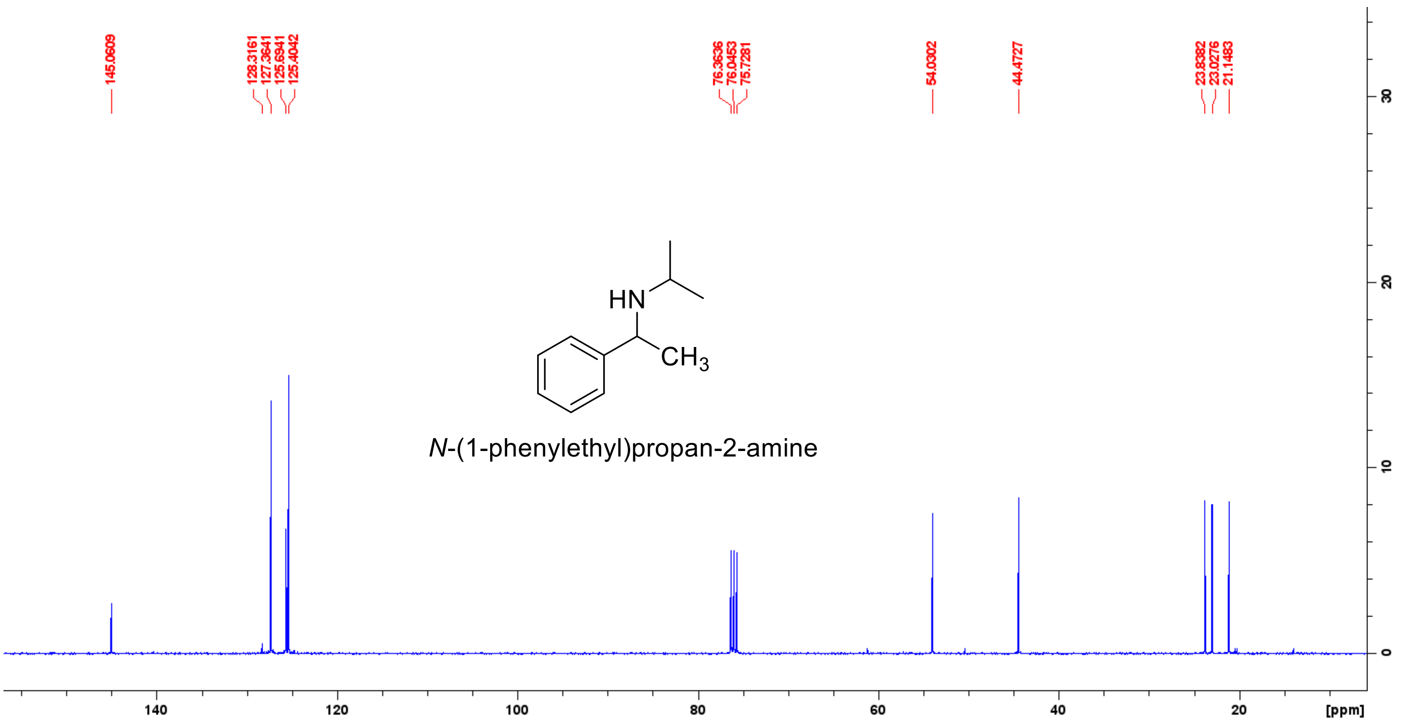

Figure S20. ^{13}C NMR spectrum for *N*-(1-phenylethyl)propan-2-amine.

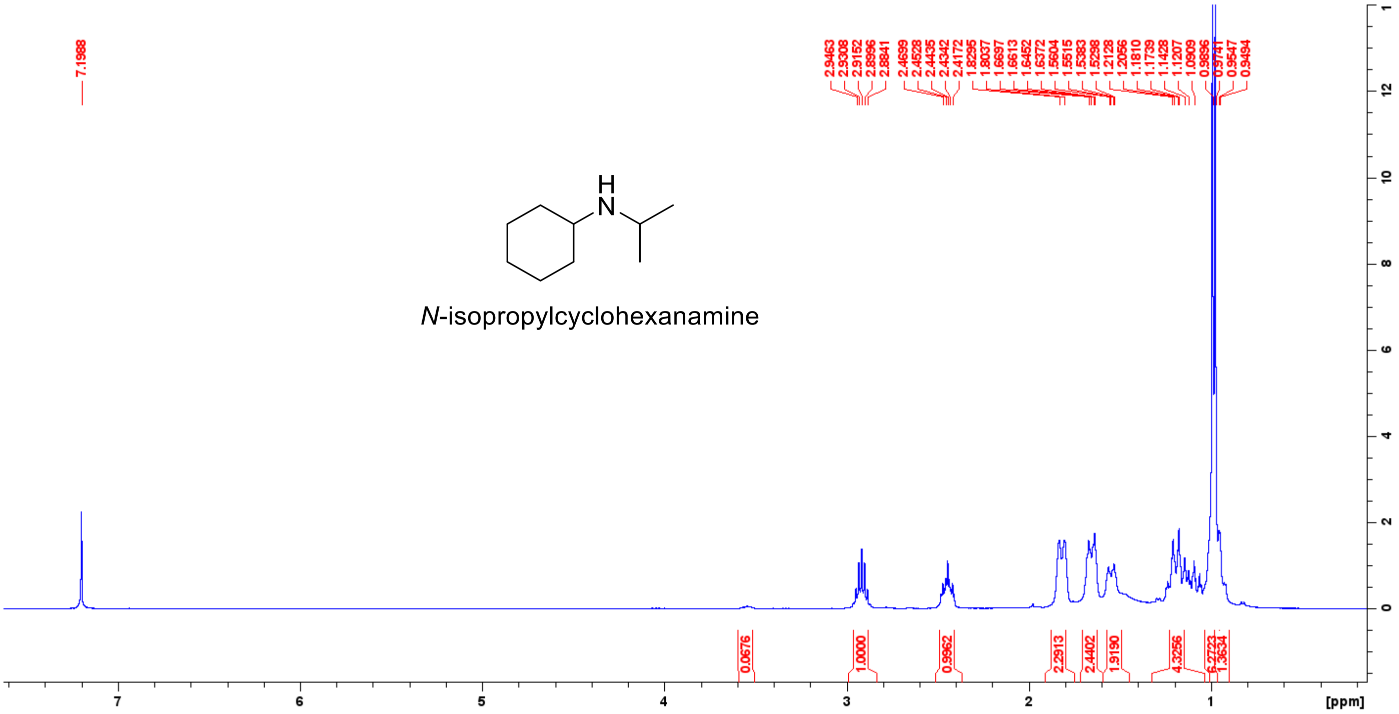

Figure S21. ^{1}H NMR spectrum for *N*-isopropylcyclohexanamine.

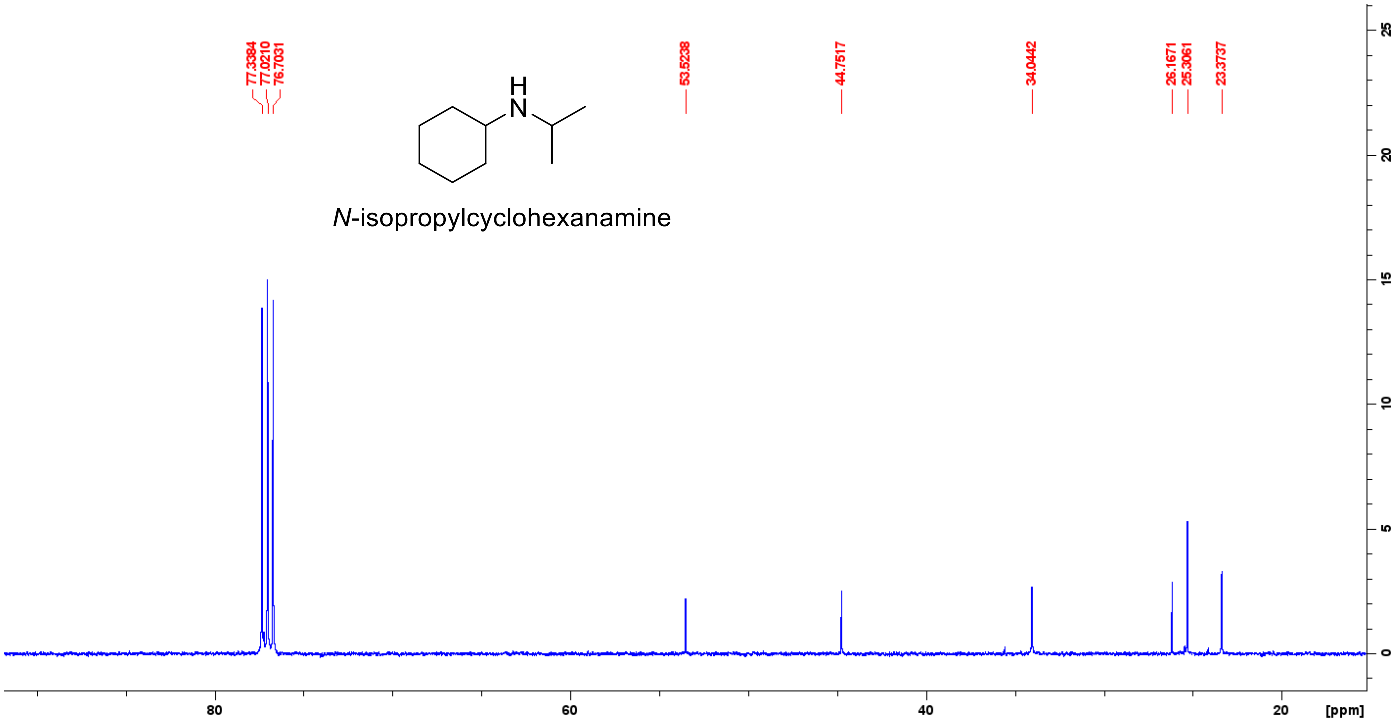

Figure S22. ^{13}C NMR spectrum for *N*-isopropylcyclohexanamine.

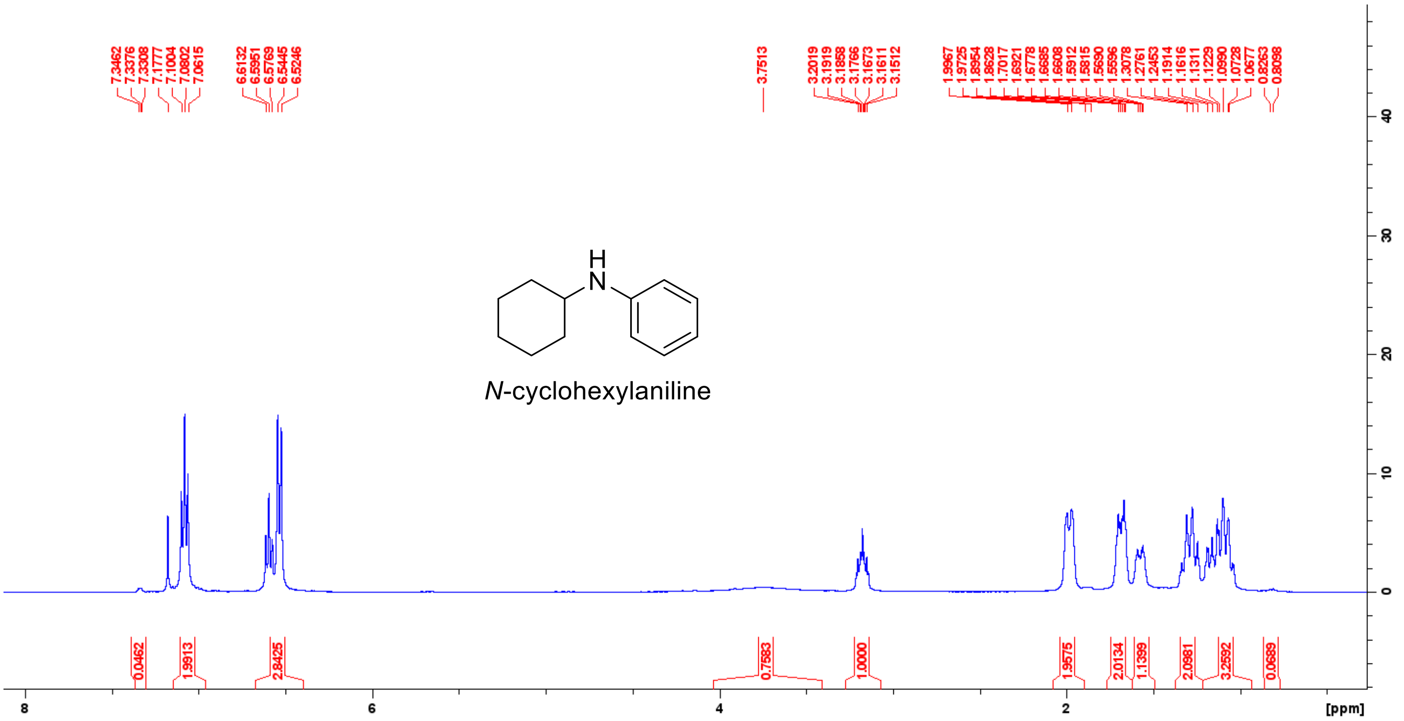

Figure S23. ^{1}H NMR spectrum for *N*-cyclohexylaniline.

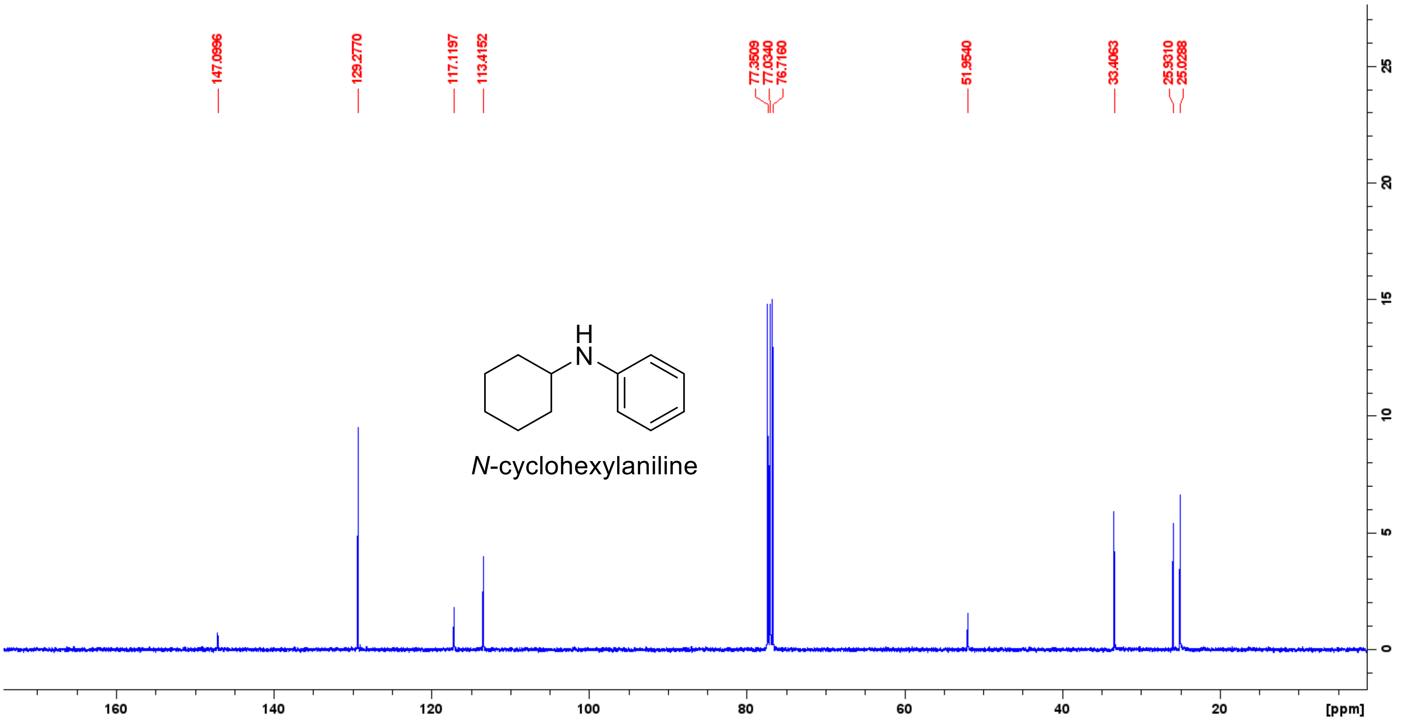

Figure S24. ^{13}C NMR spectrum for *N*-cyclohexylaniline.

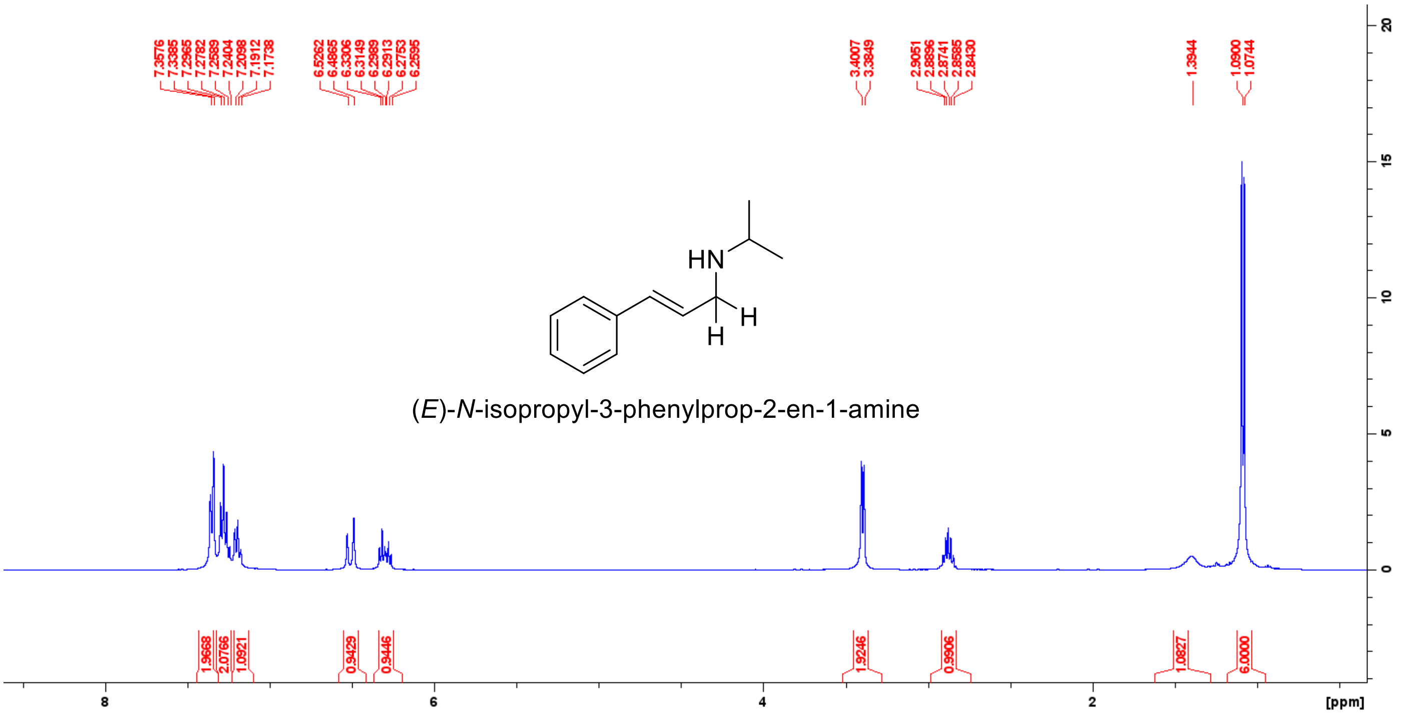

Figure S25. ^{1}H NMR spectrum for *(E)-N*-isopropyl-3-phenylprop-2-en-1-amine.

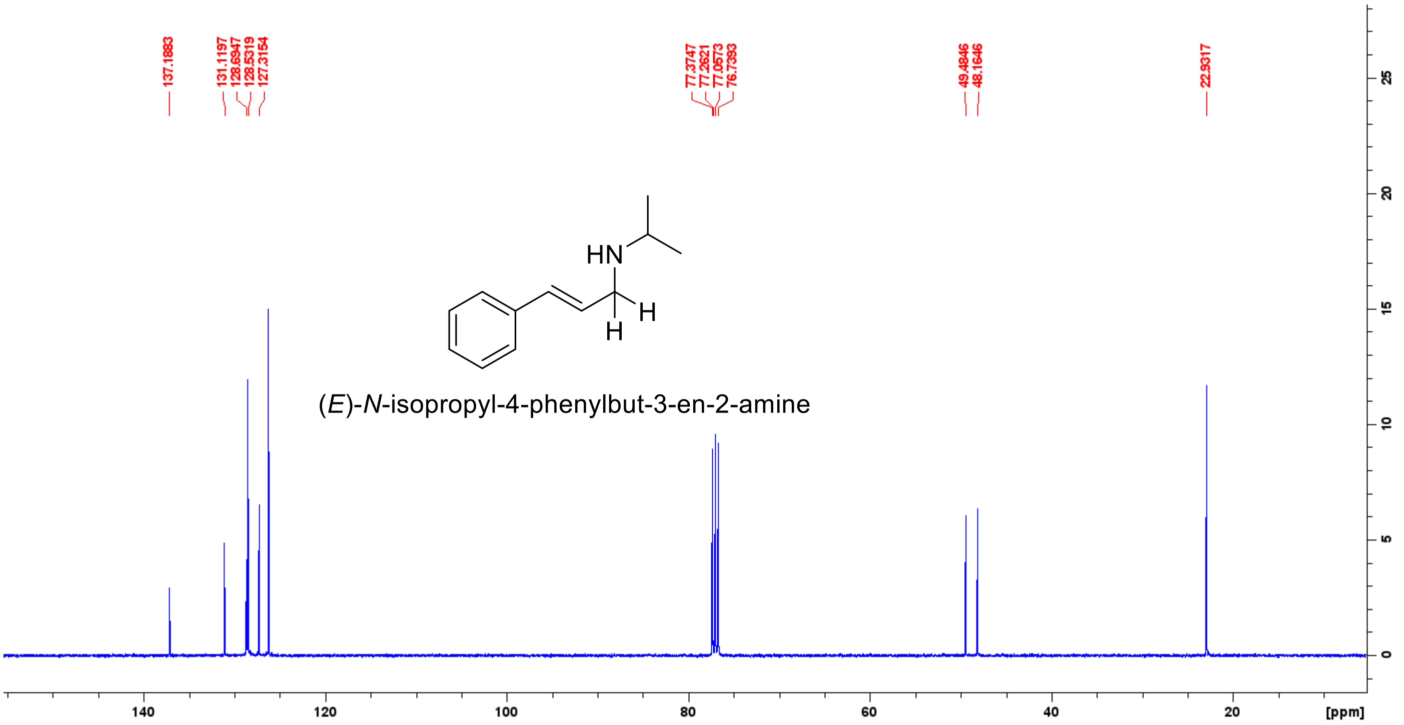

Figure S26. ^{13}C NMR spectrum for *(E)-N*-isopropyl-3-phenylprop-2-en-1-amine.

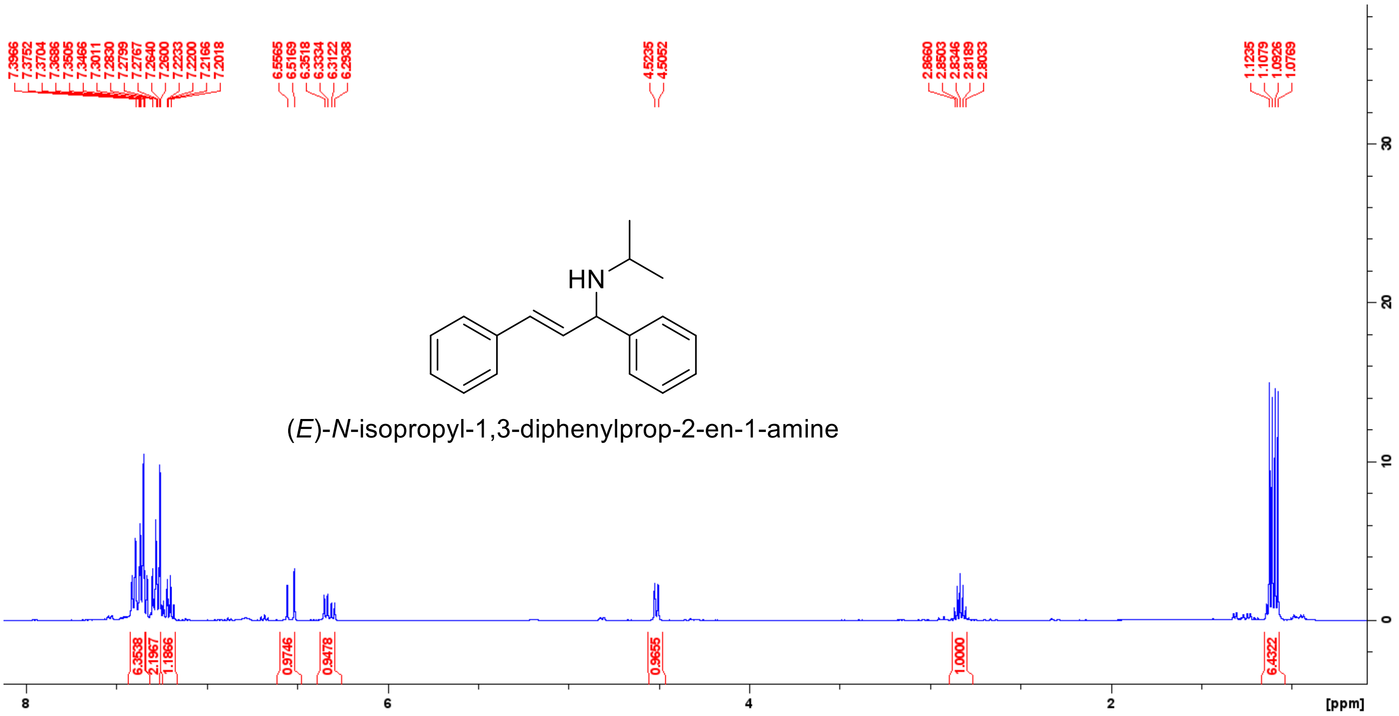

Figure S27. ^{1}H NMR spectrum for *(E)-N*-isopropyl-1,3-diphenylprop-2-en-1-amine.

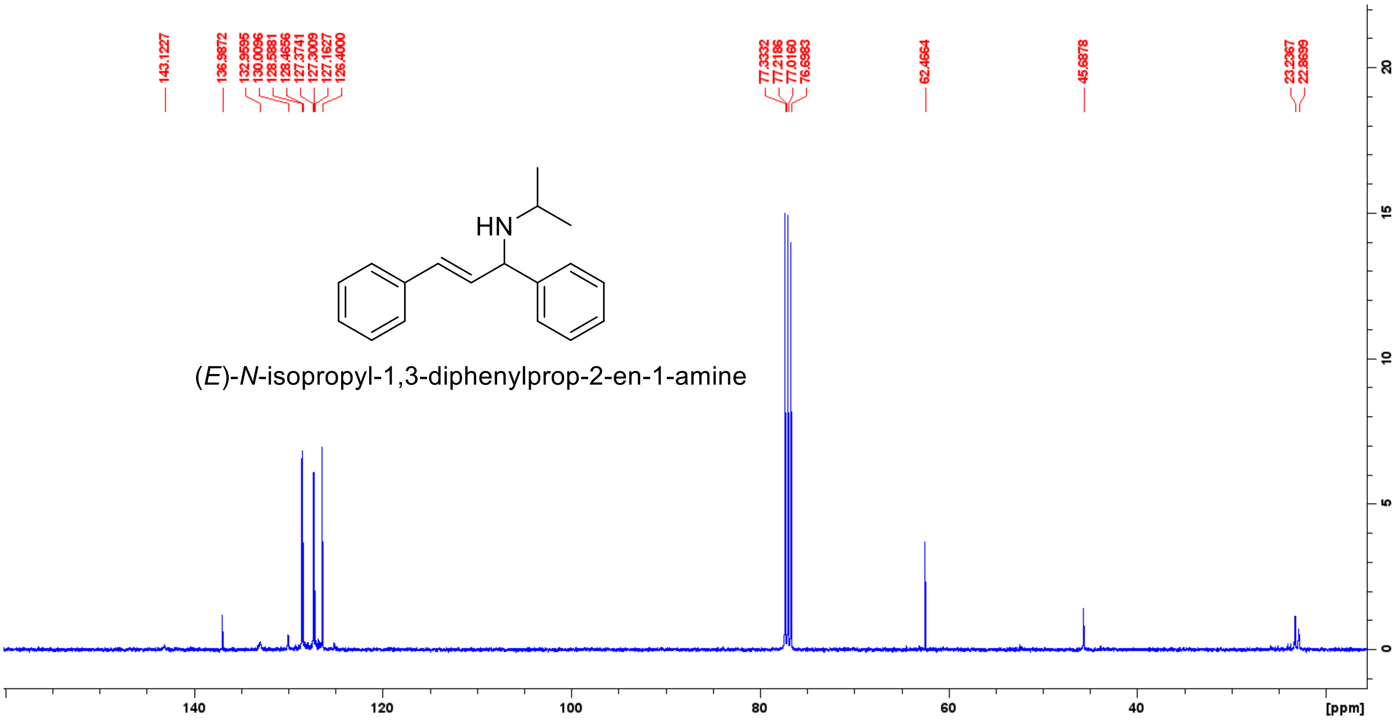

Figure S28. ^{13}C NMR spectrum for *(E)-N*-isopropyl-1,3-diphenylprop-2-en-1-amine.
